# Effective cancer immunotherapy combining mRNA-encoded bispecific antibodies that induce polyclonal T cell engagement and PD-L1-dependent 4-1BB costimulation

**DOI:** 10.3389/fimmu.2024.1494206

**Published:** 2025-01-06

**Authors:** Oana Hangiu, Rocío Navarro, Susana Frago, Laura Rubio-Pérez, Antonio Tapia-Galisteo, Laura Díez-Alonso, Marina Gómez-Rosel, Noelia Silva-Pilipich, Lucía Vanrell, Cristian Smerdou, Kenneth A. Howard, Laura Sanz, Luis Álvarez-Vallina, Marta Compte

**Affiliations:** ^1^ Department of Antibody Engineering, Leadartis SL, Tres Cantos, Madrid, Spain; ^2^ Cancer Immunotherapy Unit (UNICA), Department of Immunology, Hospital Universitario12 de Octubre (H12O), Madrid, Spain; ^3^ Immuno-Oncology and Immunotherapy Group, Instituto de Investigación Sanitaria 12 de Octubre (imas12), Madrid, Spain; ^4^ H12O-CNIO Cancer Immunotherapy Clinical Research Unit, Spanish National Cancer Research Centre (CNIO), Madrid, Spain; ^5^ Division of DNA and RNA Medicine, CIMA Universidad de Navarra, Pamplona, Spain; ^6^ Navarra Institute for Health Research (IDISNA) and Cancer Center Clínica Universidad de Navarra (CCUN), Pamplona, Spain; ^7^ Nanogrow Biotech, Montevideo, Uruguay; ^8^ Interdisciplinary Nanoscience Center (iNANO), Department of Molecular Biology and Genetics, Aarhus University, Aarhus, Denmark; ^9^ Molecular Immunology Unit, Biomedical Research Institute Hospital Puerta de Hierro, Majadahonda, Madrid, Spain

**Keywords:** T-cell engager, costimulatory antibody, cancer immunotherapy, mRNA encoded bispecific antibodies, combined RNA

## Abstract

**Background:**

Immune checkpoint inhibitors have revolutionized cancer therapy, but many patients fail to respond or develop resistance, often due to reduced T cell activity. Costimulation via 4-1BB has emerged as a promising approach to enhance the effector function of antigen-primed T cells. Bispecific T cell-engaging (TCE) antibodies are an effective way to provide tumor-specific T cell receptor-mediated signaling to tumor-infiltrating lymphocytes. mRNA-based delivery of bispecific antibodies, offer a novel approach to enhance tumor-specific immune responses while minimizing adverse effects.

**Methods:**

Two bispecific antibodies were generated: the EGFR x CD3 TCE antibody (LiTE) and the PD-L1 x 4-1BB costimulatory antibody (LiTCo), which was further fused to a high FcRn albumin variant (Albu-LiTCo). The mRNA encoding these bispecific antibodies contains an N1-methylpseudouridine modified nucleoside and regulatory sequences to ensure proper expression and stability. A series of *in vitro* assays and cell-based analyses were performed to characterize both antibodies. The *in vivo* efficacy of the mRNA-encoded bispecific antibodies was evaluated in xenograft tumor models expressing EGFR.

**Results:**

We investigated the combined effect of two mRNA-encoded Fc-free bispecific antibodies with complementary mechanisms of action: an EGFR-targeting TCE and a half-life extended PD-L1 x 4-1BB costimulatory antibody. The mRNAs encoding both bispecific LiTE^RNA^ and Albu-LiTCo^RNA^, showed similar binding specificity and *in vitro* function to their protein analogues. Pharmacokinetic studies demonstrated sustained expression of both bispecific antibodies following intravenous administration of the mRNAs formulated using a polymer/lipid-based nanoparticle (LNP) but different pharmacokinetic profiles, shorter for the TCE and longer for the PD-L1 x 4-1BB. When administered as a mRNA-LNP combination (Combo^RNA^), the growth of EGFR-positive tumors in immunocompetent mice was significantly inhibited, resulting in tumor regression in 20% of cases with no associated toxicity. Histological analysis confirmed increased T cell infiltration in the tumors treated with LITE^RNA^ and Combo^RNA^. Repeated administration resulted in sustained production of bispecific antibodies with different exposure cycles and potent antitumor activity with a favorable safety profile.

**Conclusions:**

These results highlight the potential of combining two mRNA-encoded bispecific antibodies with different mechanisms of action and programmable half-life for cancer immunotherapy.

## Introduction

Immune checkpoint inhibitors (ICIs) unleash the immune system’s full antitumor potential for robust responses (1). However, a significant proportion of cancer patients do not respond or develop resistance over time (2). Reduced functional activity of tumor infiltrating lymphocytes (TILs) and/or PD-1/PD-L1 expression are factors contributing to response heterogeneity (3). Agonistic antibodies to co-stimulatory receptors, such as 4–1BB (CD137), have emerged as a promising strategy to enhance T cell function and have demonstrated synergistic effects with ICIs, improving antitumor responses in preclinical studies (4, 5). 4-1BB is a member of the tumor necrosis factor receptor (TNFR) superfamily that is expressed on antigen-activated T cells but not on resting T cells, which could restrict stimulation to tumor-reactive T cells (6–8). In preclinical models, anti-4–1BB agonistic mAbs restored CD8^+^ T cell function and promoted antitumor responses (9). However, off-tumor toxicities have significantly hindered the clinical development of anti-4-1BB agonistic IgGs (10–12). The anti-human 4-1BB human IgG4 urelumab (BMS-663513) demonstrated clinical activity but caused fatal liver toxicity (13, 14). Several studies suggest that toxicity is likely dependent on further crosslinking provided by Fc-Fc gamma receptor (FcγR) interaction (10, 11, 15, 16). New therapeutic strategies aim to limit 4-1BB costimulation to the tumor microenvironment. These approaches include Fc-free or Fc-silent bispecific antibodies targeting 4-1BB as well as tumor or stromal cells (10, 17–21). Previous studies from our research group have demonstrate lack of liver toxicity associated with other Fc-less constructs derived from the same anti-4-1BB moiety (10, 22), in contrast to the off-tumor toxicities observed in rodents treated with anti-4-1BB IgG. Several clinical trials combining such costimulatory antibodies with anti-PD-1/PD-L1 moieties are ongoing, and preliminary data suggest that these strategies are well tolerated, enhance TIL functions, and exhibit antitumor efficacy, supporting further investigation in advanced solid tumors (3, 23–25).

Beyond immune checkpoint blockade and costimulatory agonists, bispecific T cell-redirecting antibodies have gained momentum in recent years, with ten T cell engagers (TCE) in the market as of December 2024. TCE simultaneously bind to a tumor-associated antigen (TAA) and to CD3 in the TCR complex, redirecting T cells to kill tumor cells regardless of TCR specificity (26–29). However, potent T cell responses can result in off-target effects and systemic cytokine release-associated toxicities (30). Various Fc-free formats such as the diabody, the tandem single-chain variable fragment (ta-scFv), and the tandem of a single-domain antibody (V_HH_) and a scFv (LiTE, *Light T cell Engager*) have been developed to enhance their efficacy and reduce side effects (31, 32). TCE have shown great potential in hematological cancers, with seven FDA (Food and Drug Administration)-approved bispecific antibodies for the treatment of leukemia, non-Hodgkin lymphoma (NHL), and multiple myeloma (33, 34). However, their efficacy in solid tumors is still rather limited (35). In May 2024, the Food and Drug Administration (FDA) granted accelerated approval for tarlatamab, a DLL3xCD3 half-life-extended (HLE) TCE with a silenced Fc region for small cell lung cancer (SCLC) (36). Alternatively, TAA-targeted HLE Fc-free bispecifics have been generated by fusion to an engineered human serum albumin variant with high binding (albumin^HB^) to human FcRn^29^ such as the Albu-LiTE (37) and the 4-1BB agonist Albu-LiTCo (*Light T cell Costimulatory)* antibody (22).

Another strategy to avoid frequent dosing of Fc-free bispecific antibodies while overcoming problems associated with recombinant protein production is the use of mRNA. The advantages of mRNA-based gene delivery over viral vectors and plasmid DNA have led to its increased popularity in numerous therapeutic areas (38, 39). Synthetic mRNA allows for rapid and transient protein production without nuclear entry, avoiding genome integration. A preclinical study demonstrated that intravenous administration of mRNA encoding Fc-free bispecific TAAxCD3 sustained functional antibody production and resulted in tumor regression in mice (40). In addition, the first phase 1 clinical trial of mRNA-encoded TCE demonstrated safety and the potential to achieve therapeutic levels of circulating antibodies (41).

In the present study, we generated mRNA-encoded EGFRxCD3 LiTE and PD-L1x4-1BB Albu-LiTCo. Both mRNAs promoted EGFR-specific tumor cell lysis and PD-L1 -dependent costimulatory activity *in vitro*, respectively, and exhibited favorable pharmacokinetic properties *in vivo*. In addition, the combination of LiTE^RNA^ and Albu-LiTCo^RNA^ enhanced T cell activation and tumor cell lysis and significantly delayed tumor growth in immunocompetent mice, offering a promising approach for cancer immunotherapy.

## Materials and methods

### Mice

Seven-week-old female BALB/c were purchased from Envigo (Envigo Rms Spain S.L). Animals were housed in controlled conditions of temperature (21 ± 1°C), humidity (50 ± 5%), and 12 hours light and dark cycles. Animals were maintained under specific-pathogen-free conditions, and sterilized water and food were available *ad libitum*. All animal procedures were performed in accordance with European Union Directive 86/609/EEC and Recommendation 2007/526/EC, enforced in Spanish law under RD 1201/2005. Animal protocols were performed in strict adherence to the guidelines stated in the International Guiding Principles for Biomedical Research Involving Animals, established by the Council for International Organizations of Medical Sciences (CIOMS) and were approved by the Ethics Committee of Animal Experimentation of the Instituto Investigación Sanitaria Puerta de Hierro-Segovia de Arana (Hospital Universitario Puerta de Hierro Majadahonda, Madrid, Spain). Procedures were also approved by the Animal Welfare Division of the Environmental Affairs Council of the Government of Madrid (076/19).

### Cells and culture conditions

HEK293 (CRL-1573), CT26 (CRL-2638) and CHO-K1 (CCL-61) were obtained from American Type Culture Collection (ATCC) (Rockville, MD, USA). Mouse CT26 cells (CRL-2638) infected with p-BABEpuro-hEGFR expressing human EGFR (CT26^EGFR^) were provided by Dr M. Rescigno (42) (European Institute of Oncology, Milan). Production of lentiviral vectors for the generation of CT26 and CT26^EGFR^ cells expressing the firefly luciferase (Luc) gene has been described previously (43). CHO-K1 cells stably expressing human PD-L1 (CHO^PD-L1^) were obtained from Genlantis (xCELLerateTMPD-L1 Stable Cell Line, XCL-PDL1) and CHO-K1 cells stably expressing human EGFR (CHO^EGFR^), or both (CHO^PD-L1/EGFR^) were generated using hEGFR encoding commercial lentiviral particles (G&P Biosciences, cat# LTV0169). Both cell lines were infected overnight at a final multiplicity of infection (MOI) of 10. Cells were supplemented with 500 µg/ml of Hygromycin B (Sigma-Aldrich) or 500 µg/ml of Hygromycin B+100 µg/ml of Blasticidin S HCl (Thermo Fisher Scientific), respectively. HEK293 cells expressing mouse 4-1BB (HEK293^4-1BB^) were obtained from Prof I. Melero (CIMA, Pamplona, Spain). The cells were grown in Dulbecco’s modified Eagle’s medium (DMEM) supplemented with 2 mM L-glutamine (Lonza, USA, cat# BE17-605E), 10% (vol/vol) heat inactivated fetal bovine serum (FBS) (Gibco, USA, cat# 10500-064) and antibiotics (100 units/mL penicillin, 100 mg/mL streptomycin) (Gibco, USA, cat# 15140163), referred to as DMEM complete medium (DCM), at 37 °C in 5% CO_2_ humidity. T cell line, Jurkat, clone E6-1 (Human acute T cell leukemia; TIB-152) was obtained from American Type Culture Collection (ATCC) and T cell line 2B4 (Mouse T-cell hybridoma; CVCL_4Z38) was provided by Prof. J. R. Regueiro (UCM, Madrid, Spain). Cells were grown in Roswell Park Memorial Institute medium (RPMI) supplemented with 2 mM L-glutamine, 10% (vol/vol) heat inactivated FBS, and antibiotics (100 units/mL penicillin, 100 mg/mL streptomycin), referred to as RPMI complete medium (RPMI-C), at 37 °C in 5% CO_2_ humidity. The cell lines were routinely screened for mycoplasma contamination by PCR using the Mycoplasma Gel Detection Kit (Biotools, Spain, cat# 90.021-4542).

### Molecule design, expression, and purification of bispecific antibodies

The FLAG-strepII-EGa1-2C11 DNA construct was created by obtaining the anti-EGFR V_HH_ EGa1 from a previously published anti-EGFRxanti-CD3 LiTE (44), and the mouse anti-CD3ϵ V_H_ and V_L_ sequences of 2C11 scFv (45), were synthesized by GeneArt. Sequences were inserted into the pCR3.1-EGa1 expression vector using *Not*I*/*Bgl*II* restriction enzymes, resulting in the anti-EGFR x anti-mouse CD3ϵ bispecific antibody, hereafter referred to as LiTE (*Light T cell Engager*). The 6P V_HH_ anti-PD-L1 DNA sequence (46) was inserted into the pCR3.1-EGa1 expression vector using *HindIII/NotI* restriction enzymes. The DNA sequence FLAG-strepII-6P-1D8, from GeneArt, was subcloned as *Age*I/*SnaB*I into the vector containing the high affinity FcRn binding human albumin (Albumin^HB^) (47) resulting in the FLAG-strepII-6P-1D8-Albumin^HB^ construct, anti-m/hPD-L1 x anti-m4-1BB bispecific antibody, referred to as Albu-LiTCo (*Light T cell Costimulatory*). All the sequences were verified using primers FwCMV and RvBGH (**Supplementary Table 1**). Constructed vectors were stably transfected into HEK293 cells using Lipofectamine 3000 (Invitrogen, USA, cat# L3000001) and selected with 500 μg/ml Geneticin (G418) to generate stable HEK293-LiTE and HEK293-Albu-LiTCo cell lines. Antibodies were purified from free-cell culture by affinity chromatography Strep-Tactin^®^ purification system (IBA Lifesciences, Germany) using an ÄKTA Go system (Cytiva). Purified antibodies were dialyzed overnight at 4°C against PBS with 150 mM NaCl at pH 7.0. (10-20% Tris-Glycine gel) and analytical size exclusion chromatography (SEC) (Superdex 200 increase 10/300 GL; Cytiva Spain S.L.U.) were performed to determine the size and the purity of the bispecific antibodies.

### mRNA-encoding bispecific antibodies and polymer/lipid-based formulation

The template for mRNA IVT (*in vitro* transcription) was carried out by GenScript Biotech (Netherlands). The mRNA-encoding bispecific antibodies incorporates a 5’ type I capping structure (5’ Cap: Cap1) and N1-Methylpseudouridine modified nucleosides (N1-me-Ψ), to increase stability and reduce its immunogenicity (48). The IVT template (linearized plasmids), contains a 5’UTR, Kozak sequence, 3’UTR and a Poly(A)tail containing more than 110 successive adenines and the coding sequence. For *in vitro* and *in vivo* studies, mRNA-encoded LiTE (LiTE^RNA^) and mRNA-encoded Albu-LiTCo (Albu-LiTCo^RNA^) were formulated with the polymer/lipid-based formulation *Trans*IT™-mRNA Transfection Kit (Mirus Bio, USA, cat# MIR2304), in accordance with the manufacturer’s protocol. Briefly, HEK293 cells cultured in DCM were seeded at 3 × 10^5^ cells/well on a 6-well plate. The day after, LiTE^RNA^ and Albu-LiTCo^RNA^ (1 µg each) were diluted in 50 µl of Opti-MEM, followed by the addition of 2 µl of TransIT Boost reagent and 2 µl of TransIT-mRNA reagent. After two minutes of incubation, the reagent mixture complexes were added to the cell culture. The transfection was performed in duplicate, and after 48 hours incubation at 37°C, 5% CO2, the cell culture supernatants were harvested by centrifugation (2000 rpm, 5 min), pooled, and stored at -20°C until analysis. An EGFP mRNA (EGFP^RNA^) (GenScript; # SC2325-1mg) which incorporates a 5’ Cap: Cap1 structure and N1-me-Ψ, as the mRNA encoding bispecific antibodies, was used as a control. Regarding *in vivo* experiments, a modification was made to the protocol originally proposed by Stadler et al. (40) as follows. 10 micrograms of mRNA were diluted in 171.60 µl of DMEM with 4.5 g/l glucose (Thermo Fisher) and mixed with 11.20 µl of TransIT-mRNA reagent and 7.20 µl of TransIT Boost reagent. mRNA reactions were immediately vortexed, incubated for two minutes at room temperature (RT), and injected into mice within five minutes. 200 µl was the total volume of 10 µg of mRNA complex.

### Western blotting

Protein samples were separated under reducing conditions on 10–20% Tris-glycine gels and transferred to nitrocellulose membranes and probed either with anti-FLAG (clone M2; Merck KGaA, Germany, cat# F3165) monoclonal antibody (mAb) (1 μg/ml), followed by incubation with goat anti-mouse DyLight 680 (1:5000 dilution) (Rockland, cat# 800-656-002), or Horseradish peroxidase (HRP)-conjugated MonoRab™ Rabbit Anti-Camelid V_HH_ Cocktail antibody (anti-V_HH_ Cocktail mAb) (GenScript, cat#A02016) at 1:2500 dilution working dilution. Visualization of protein bands was performed with the Amersham ImageQuant™ 800 (Cytiva). Images were analyzed with Image Studio Lite software.

### ELISA

Maxisorp immunoplates (NUNC Brand Products, Denmark, cat# 439454) were coated overnight at 4°C with human (h) EGFR-hu IgG1 Fc chimera (hEGFR) (Bio-Techne R&D Systems; cat# 344-ER), mouse (m) 4-1BB-hIgG1 Fc chimera (m4-1BB) (SinoBiological Europe GmbH, Germany, cat# 50811-M02H), recombinant mPD-L1 (mPD-L1) (clone B7-H1) or recombinant hPD-L1 (hPD-L1) (clone B7-H1) (Bio-Techne R&D Systems; cat# 1019-B7 and cat# 156-B7) at 3 µg/ml in PBS. Plates were washed and blocked with 5% bovine serum albumin (BSA) or 5% nonfat dry milk (NFDM) in PBS, and 100 μl of supernatant from transfected HEK293 cells or purified antibodies at 3-fold serial dilutions, were added in triplicates and incubated for 1 hour at room temperature. The wells were washed three times with PBS + 0.05% Tween_20_, followed by three washes with PBS. For detection, anti-FLAG mAb (1μg/ml) was added for 1 hour incubation at room temperature. The plates were washed as above described, and 100 μl of HRP-conjugated goat anti-mouse (GAM) (Jackson ImmunoResearch Europe Ltd., UK, cat# 115-085-166) or HRP-conjugated sheep anti-human serum albumin (HSA-HRP) (Abcam, UK, cat# ab8941), were diluted at a working concentration of 1:2000 in PBS + 1% BSA or, PBS + 1% NFDM, respectively.

The functional expression of LiTE^RNA^ and Albu-LiTCo^RNA^ in supernatants were assayed by coating MaxiSorp plates with hEGFR, m4-1BB, and mPD-L1 (3 µg/ml) in PBS overnight at 4°C. After washing and blocking with 5% BSA or 5% NFDM, duplicates of supernatants were added at room temperature for 1 hour. Plates were incubated for 1 hour at room temperature with HRP-conjugated anti-V_HH_ Cocktail (1:2000) diluted in PBS + 0.01% Tween_20_, or HSA-HRP (1:2000) diluted in PBS + 1% NFDM for detection. The estimated concentration of LiTE^RNA^ (3.3 µg/ml) and Albu-LiTCo^RNA^ (3.9 µg/ml) was determined using the same procedure, with 3-fold serial dilutions of purified antibodies (as standard controls) and detection by HRP-conjugated anti-V_HH_ cocktail (1:2000). Plates were washed and developed using Tetramethylbenzidine (TMB) (Merck KGaA cat# T0440), stopped with 4N H_2_SO_4_, and measured at 450-570 nm using a Bio-Rad microplate-reader. Data was analyzed and plotted in GraphPad Prism 8.4.0.

### Flow cytometry

HEK293, HEK293^4-1BB^, CHO, CHO^PD-L1^, CT26, CT26^EGFR^, Jurkat or 2B4 cells (2.5 x 10^5^ cells/well) were incubated on ice for 30 min with conditioned media from transfected HEK293 cells or purified antibodies (5 μg/ml), washed and incubated with anti-FLAG mAb (5 μg/ml) for 30 min and detected with a R-phycoerythrin (PE) F(ab’)_2_ Fragment Goat Anti-Mouse (GAM-PE) IgG, Fcγ fragment specific (Jackson ImmunoResearch, cat# 115-116-071) at 1:500 working dilution. Anti-4-1BB IgG (clone 3H3; BioXcell; cat# BE0239), anti-PD-L1 IgG (atezolizumab), anti-EGFR IgG (cetuximab), and anti-CD3 IgG (clone 145-2C11; Biolegend, USA, cat# 100303) were used as controls, at 5 μg/ml. Binding was detected after incubation with R-Phycoerythrin (PE) F(ab’)_2_ Fragment Goat Anti-Human (GAH) IgG, Fcγ fragment specific (Jackson ImmunoResearch, cat# 109-116-170) or GAM-PE at 1:500 working dilutions, as needed.

The binding analysis of LiTE^RNA^ and Albu-LiTCo^RNA^ was also performed using the anti-FLAG mAb followed by GAM-PE at a working dilution of 1:500. The cell surface expression of mPD-L1 was analyzed on CT26 and CT26^EGFR^ cells, before and after 24 hours incubation with soluble recombinant human IFNγ (20 ng/ml) (#300-02, PeproTech) at 37°C. Cells were stained with PE-conjugated anti-mPD-L1 mAb (clone 10F.9G2, cat# 124308 BioLegend) (2 μg/ml). A minimum of 20,000 events were acquired for each sample with a FACSCanto II cytometer (BD Biosciences). Collected data was evaluated using the FlowJo X (BD Biosciences) software.

### Antigen specific T cell activation assay

CT26 and CT26^EGFR^ cells (0.3 x 10^5^ cells/well) were plated in triplicate in flat-bottom 96-well plates overnight at 37°C. Total splenocytes from 8-week-old female BALB/c mice were resuspended in RPMI supplemented with 10% FBS and 50 μM β-mercaptoethanol (Life Technologies) and added to the plate (1.5 x 10^5^ cells/well) at a effector/target ratio of 5:1. LiTE and purified anti-mouse CD3ε IgG (clone 145-2C11) were added at 3.34 nM (equivalent to 0.175 µg/mL and 0.5 µg/mL respectively) equimolar concentration. After 24 hours supernatants were collected and assayed for IFNγ and IL-2 (Diaclone, France, cat# 861.050.005, and cat# 861.000.005) secretion by ELISA following manufacturer’s protocol. Cells were collected and incubated on ice for 30 min with fluoresceine (FITC) conjugated-Hamster anti-mouse CD69 IgG_1_ (clone H1.2F3, cat# 561929, BD Biosciences, USA) or FITC-conjugated Hamster IgG_1_, Isotype Control (clone G235-2356, cat# 553953 BD Biosciences) (3 μg/ml), both diluted in PBS + 1%FCS and washed. Samples were analyzed with FACSCanto II cytometer and FlowJo X software was used for analyzing flow cytometry data.

### Cytotoxicity assay

CT26 and CT26^EGFR Luc^ (0.3 x 10^5^ cells/well) gene-modified for the expression of luciferase, were seeded in triplicate in flat-bottom 96-well plates overnight at 37°C. Next day, target cells were incubated with increasing amounts of LiTE (0 to 600 nM) or LiTE^RNA^-HEK293 undiluted supernatant (3.3 µg/ml). Total splenocytes were isolated from 8-week-old female BALB/c mice, resuspended in RPMI supplemented with 10% FBS and were added to the plate (1.5 x 10^5^ cells/well) at 1:5 target:effector ratio. After 48 hours of incubation, D-luciferin substrate (20 μg/well, Promega E1602) was added, and relative light units (RLU) were measured with the luminescence plate reader Infinite 1200 (Tecan Trading). Percent tumor cell viability was calculated by dividing the mean bioluminescence of each sample by the mean bioluminescence of the input number of control target cells, multiplied by 100. Specific lysis is the difference in tumor cell viability relative to control (0%). Wells with target and effector cells in the absence of antibodies were set as 100% of viability.

### Neutralization of PD-1/PD-L1 interaction by competition ELISA

Potency to neutralize PD-L1 binding to PD-1 was assessed in competition ELISA where 4 µg/ml recombinant hPD1-hIgG1 Fc chimera (hPD-1) (SinoBiological Europe GmbH, cat# 10377-H02H) was coated. Three-fold serial dilutions of anti-PD-L1 IgG (atezolizumab) or purified Albu-LiTCo were simultaneously incubated with 1 μg/ml of recombinant biotinylated hPD-L1/B7-H1 Protein His-tagged (SinoBiological Europe GmbH, cat# 10084-H08H-B) for 1 hour at room temperature, washed and detected by streptavidin-HRP (Sigma-Aldrich, cat# RABHRP3) (1:1000) followed by TMB. The reaction was stopped with 4N H_2_SO_44_ and the absorbance was measured at 450-570 nm using a Bio-Rad microplate-reader. Data was analyzed and plotted using IC50 values calculated by fitting the concentration–response curves with a four-parameter logistic regression model using GraphPad Prism 8.4.0 software.

### Blockade bioassay PD-1/PD-L1 interaction

Jurkat T cells stably expressing human PD-1 and NFAT-induced luciferase (JurkatNFAT-RE−*luc*/PD-1, referred as Jurkat^PD-1^) and CHO-K1 cells stably expressing human PD-L1 (PD-L1 aAPC/CHO-K1) were obtained from Promega (cat# J1250) and were used following the manufacturer’s instructions. Briefly, 0.3 × 10^5^ PD-L1 aAPC/CHO-K1 cells/well were seeded in 96-well white flat-bottom plates (Merck KGaA, cat# CLS3922) in DCM and incubated overnight at 37 °C. Next day, medium was removed and anti-PD-L1 IgG or Albu-LiTCo were added equimolarly at two-fold serial dilutions in 30 µl of RPMI+ 1% FBS/well. Then, 1.5 × 10^5^ Jurkat^PD-1^ cells/well were added in 30 µl RPMI + 1% FBS and incubated for 6 hours at 37°C. Bio-Glo™ Luciferase Assay Reagent (Promega; cat# G7941) was added and luciferase activity was assessed using a Tecan Infinite F200 plate-reading luminometer (Tecan Trading). The experiments were performed in triplicates and data are reported as x-fold of induction relative to the values obtained from unstimulated cells. Data was analyzed and plotted using GraphPad Prism 8.4.0 software.

### Antigen-specific T cell costimulatory assay

For studies with target cells, CHO and CHO^PD-L1/EGFR^ cells (0.3 x 10^5^ cells/well) were seeded overnight at 37°C and 5% CO_2_. Mouse CD8a^+^ T cells were purified from the spleens of 8-week-old BALB/c female mice using the mouse CD8a^+^ T Cell Isolation Kit (Miltenyi Biotec, Germany, cat# 130-095-236), resuspended in 100 µl of culture media (RPMI supplemented with 10% FBS and 50 μM β-mercaptoethanol (Life Technologies) and pre-activated with soluble LiTE or anti-CD3ε IgG (clone 145-2C11; Biolegend, cat# 100303) at 3.34 nM equimolar final concentration (0.175 µg/mL and 0.5 µg/mL, respectively) for at least 5 hours at 37°C. Mouse CD8a^+^ T cells were added to the plate (1.5 x 10^5^ cells/well) at an effector/target ratio of 5:1, along with purified Albu-LiTCo or anti-4-1BB IgG (clone 3H3) diluted in 100 µl of culture media at a final concentration of 6.67 nM equimolar dose. As a negative control for costimulation, LiTE-pre-activated mouse CD8a+ T cells were added to the co-culture in the absence of both Albu-LiTCo and anti-4-1BB IgG. Additionally, non-pre-activated mouse CD8a+ T cells were included in the co-culture as a negative control for the assay. For studies with purified antigens, 96-well flat-bottom plates were coated with 3 µg/ml of recombinant mPD-L1/B7-H1 hIgG1 Fc chimera (R&D, cat# 1019-B7-100#) or BSA as a control, overnight at 4 °C. After washing, plates were blocked with RPMI + 5% BSA for 1 h at 37°C. Mouse CD8a^+^ T cells were isolated as described above, resuspended in 100 µl of culture media and pre-activated with anti-CD3ε IgG (3.34 nM) for at least 5 hours at 37°C. Mouse CD8a+ T cells (1.5 x 10^5^ cells/well) were then added to the plates with Albu-LiTCo, anti-4-1BB IgG or purified mouse IgG_1K_ isotype (clone MG1-45; Biolegend, cat# 401402) diluted in 100 µl of culture media at a final concentration of 6.67 nM equimolar dose. As a negative control for costimulation, CD3-pre-activated mouse CD8a^+^ T cells were added to the plates without Albu-LiTCo, anti-4-1BB IgG or IgG_1K_ isotype. Additionally, non-pre-activated mouse CD8a+ T cells were included as a negative control for the assay.

For the functional analysis of LiTE^RNA^-HEK293 supernatants (LiTE^RNA^), CHO and CHO^PD-L1/EGFR^ cells (0.3 x 10^5^ cells/well) were seeded in 96-flat well plates overnight at 37°C. Mouse CD8a^+^ T cells were isolated as previously described, and directly resuspended at 1.5 x 10^5^/well. in 100 µl of undiluted LiTE^RNA^ (3.3 µg/ml). Cells were pre-activated for at least 5 hours at 37°C and added to the co-culture at an E/T ratio of 5:1. Albu-LiTCo, anti-4-1BB IgG, anti-PD-L1 IgG (clone MPDL3280A; MCE, cat# HY-P9904) or IgG1K isotype were then added in 100 µl of culture medium at a final concentration of 6.67 nM equimolar dose. As a negative control for costimulation, LiTE^RNA^ pre-activated mouse CD8a+ T cells were added to the plates in the absence of the above antibodies. Additionally, non-pre-activated mouse CD8a+ T cells were included in the co-culture as a negative control for the assay.

For the functional analysis of Albu-LiTCo^RNA^-HEK293 supernatant (Albu-LiTCo^RNA^), CHO and CHO^PD-L1/EGFR^ cells (0.3 x 10^5^ cells/well) were seeded in 96-flat well plates overnight at 37°C. Mouse CD8a^+^ T cells were purified, resuspended in 100 µl of culture media (1.5 x 105 cells/well) and pre-activated with soluble LiTE at 3.34 nM equimolar final concentration for at least 5 hours at 37°C, and added to the coculture at an E/T ratio of 5:1. Afterwards, 100 µl of undiluted Albu-LiTCo^RNA^ (3.9 µg/ml) was added to the co-culture. As a negative control for costimulation, LiTE-pre-activated mouse CD8a+ T cells were added to the plates in the presence of 100 µl of EGFP^RNA^-HEK293 supernatant (EGFP^RNA^). Additionally, non-pre-activated mouse CD8a+ T cells combined with 100 µl of EGFP^RNA^ were included in the co-culture as a negative control for the assay.

In all cases, co-cultures were incubated for 72 hours, supernatants were collected and assayed for IFNγ secretion by ELISA (Diaclone, cat# 861.050.005) following manufacturer’s protocol. Results are expressed as a mean ± SD from one of at least three separate experiments. All the experiments were performed in triplicates in 200 µl final volume.

### Pharmacokinetics study

9-week-old female BALB/c mice were intravenously (i.v.) injected (tail vein) with a single dose of 10 μg mRNA-polymer/lipid (mRNA-LNP) formulated LiTE^RNA^ (*n* = 3) or, Albu-LiTCo^RNA^ (*n* = 3) or PBS (*n* = 3). A similar assay was performed with Albu-LiTCo (n=4) and LiTE (n=4) purified antibodies injected i.v. once at 1 mg/kg into 8-week-old female BALB/C mice. In both experiments, blood samples were collected at 0, 4, 24, 48, 72, 98 and 168 hours in separation gel BD microtubes (BD Biosciences, USA, cat# 365968), centrifuged at 5000 rpm for 1.5 min to obtain serum and stored at -20 °C until use. Detection and quantification of LiTE^RNA^ and Albu-LiTCo^RNA^ were determined by ELISA. Briefly, MaxiSorp plates were coated with hEGFR, m4-1BB, and mPD-L1 (3 µg/ml) in PBS overnight at 4°C. After washing and blocking with 5% BSA, duplicates of non-diluted sera were added at room temperature for 1 hour. Standards were 3-fold serial dilutions of purified recombinant LiTE or Albu-LiTCo antibodies. Plates were incubated for 1 hour at room temperature with HRP-conjugated anti-V_HH_ Cocktail (1:2000) diluted in PBS + 0.01% Tween_20_ for detection. Plates were washed and developed using (TMB), stopped with 4N H_2_SO_4_, and measured at 450-570 nm using a Bio-Rad microplate-reader. Data was analyzed and plotted in GraphPad Prism 8.4.0.

### *Ex vivo* specific tumor lysis

Undiluted LiTE- and Albu-LiTCo-containing mouse serum collected at 4, 96, and 168 hours post-mRNA-LNP administration were added in triplicates to cocultures of mouse splenocytes (1.5 x 105 cells/well) with either EGFR-positive (CT26^EGFRLuc^) or EGFR-negative (CT26^Luc^) cells (0.3 x 105 cells/well) at an E/T ratio of 5:1. Translated protein levels peaked at approximately 100 ng/ml within 4 hours. LiTE stabilized at 40 ng/ml for up to 72 hours, while for Albu-LiTCo, remained at 30 ng/ml for up to 168 hours. Total splenocytes were isolated from 8-week-old female BALB/c mice and resuspended in RPMI supplemented with 10% FBS. After 48 hours of incubation, D-luciferin substrate (20 μg/well, Promega E1602) was added, and relative light units (RLU) were measured with the luminescence plate reader Infinite 1200 (Tecan Trading). Tumor cell viability was calculated by dividing the mean bioluminescence of each sample by the mean bioluminescence of the input number of control target cells, multiplied by 100. Specific lysis is the difference in tumor cell viability relative to control (0%). Wells with target and effector cells in the absence of antibodies were set as 100% viability.

### Therapeutic studies

CT26^EGFR^ cells (1.5 x 10^6^/mouse) were implanted subcutaneously into the dorsal space of 6-week-old female BALB/c mice. At day 4 animals were randomized into treatment groups (n=5). Tumor growth was monitored by caliper measurements three times a week, measurements were conducted randomly by a blinded investigator. Mice received an intravenous (i.v.) injection of PBS, LiTE^RNA^ formulated with 10 µg polymer/lipid, Albu-LiTCo^RNA^, or Combo^RNA^ (LiTE^RNA^ and Albu-LiTCo^RNA^) through the tail vein every week for three weeks. On days 0 and 14 after treatment, mice were anesthetized and bled for serum collection, which was stored at -20°C until use. IFNγ levels in serum were analyzed by ELISA. Mice weights were measured twice a week to monitor toxicity. Mice were euthanized when tumor size reached a volume of 1.5 cm^3^, tumors ulcerated, or at signs of distress.

### Immunohistochemistry

Tumors from different treatment groups were collected and fixed in 4% formalin solution (Sigma-Aldrich) for 48 hours. After extensive washing in PBS, tissues were embedded in paraffin (FFPE). 4μm-thick FFPE sections were processed on the Dako PT Link system for optimized staining consistency. Antigen retrieval was performed with EDTA pH9, and sections were incubated with CD8 FLEX (clone C8/144B, DAKO) or Perforin (clone 5B10, Abcam) antibodies on the Dako Autostainer Link 48 platform. Nuclei were counterstained with Harris’ hematoxylin. Positive control sections were included for each staining run. All slides were dehydrated, cleared, and mounted with a permanent mounting medium for microscopic evaluation. Whole digital slides were acquired with a Zeiss AxioScan Z1 slide scanner, and positive versus total cells were automatically quantified using QuPath v0.4.3 software (49).

### Statistical analysis

Statistical analysis was performed using GraphPad Prism Software version 8.4.0. All *in vitro* experiments were done in triplicates, and values are presented as mean ± SD from one of at least three separate experiments. Significant differences (*P* value) were determined using a two-tailed, unpaired Student’s *t*-test assuming a normal distribution. P values are indicated in the corresponding figures. When no statistically significant differences were observed, the corresponding data were not displayed. IC50 and EC50 were calculated using a nonlinear regression curve (log agonist vs. normalized response-variable response). Tumor volumes for individual mice in each treatment group are plotted, and mean tumor volumes are presented for each group as mean ± SD using a scatter plot. Differences in tumor growth were determined by one-way ANOVA adjusted by the Tukey’s correction for multiple comparisons.

## Results

### Generation and characterization of bispecific antibodies

**Figure 1:**
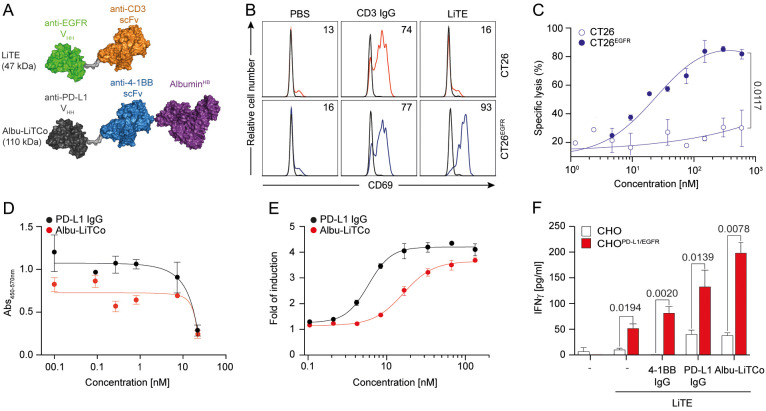
Functional characterization and mechanisms of action of LiTE and Albu-LiTCo antibodies. Schematic representation of EGFR-targeted LiTE and Albu-LiTCo antibodies **(A)**. LiTE-mediated EGFR-dependent activation of tumor cells **(B)**. CT26 and CT26^EGFR^ were cocultured with mouse splenocytes (E:T ratio 5:1) in the presence of LiTE or anti-CD3 IgG at 3.34 nM equimolar concentration. Mouse CD69 expression was measured by flow cytometry after 24 hours **(B)**. LiTE-mediated EGFR-dependent cytotoxicity of tumor cells **(C)**. Luciferase-expressing CT26 and CT26^EGFR^ cells were cocultured with splenocytes (E:T ratio 5:1) and different concentrations of LiTE. Specific lysis was measured by bioluminescence at 48 hours. Percent specific lysis was calculated relative to control. Data are mean ± SD (n = 3). Significance was calculated by unpaired Student’s *t* test. Competition ELISA for PD-L1/PD-1 interaction inhibition by anti-PD-L1 IgG or Albu-LiTCo **(D)**. Data are mean ± SD (n = 3). One representative experiment from two independent experiments is shown. LogIC50 is shown. Blocking activity of Albu-LiTCo in a cell-based bioassay **(E)**. JurkatPD-1 cells were cocultured with CHO^PD-L1^ cells and increasing concentrations of anti-PD-L1 IgG or Albu-LiTCo. Luminescence was measured after 6 hours. Data are expressed as fold induction relative to unstimulated JurkatPD-1 cells. Representative dose-concentration curves are shown as mean ± SD (n = 3). IC50 is shown. Agonistic activity of Albu-LiTCo **(F)**. Wild type CHO cells or CHO^PD-L1/EGFR^ were cocultured with LiTE-activated mouse CD8a^+^ T cells (3.34 nM) in the presence of Albu-LiTCo, anti-4-1BB IgG or anti-PD-L1 IgG at 6.67 nM equimolar dose. IFNγ secretion was determined after 72 h Negative controls (–) consisted of CD8^+^ T cells cultured with either CHO or CHO^PD-L1/EGFR^, and in the absence (- control in the left) or presence (- control in the right) of soluble LiTE. Data are mean ± SD (n = 3). One representative experiment of three independent experiments is shown. Significance was calculated by unpaired Student’s *t* test.

We generated two V_HH_ - scFv tandem bispecific antibodies, LiTE and Albu-LiTCo. The LiTE (44) was obtained by fusing the anti-mouse/human EGFR V_HH_ (EGa_1_) to the anti-mouse CD3ϵ scFv (2C11) through a flexible G_4_S linker (**Figure 1A**; **Supplementary Figures 1A, B**). The LiTCo was generated by fusing the anti-mouse/human PD-L1 V_HH_ (Nb6P) to the anti-mouse 4-1BB scFv (1D8) (10, 22) through a G_4_S linker (**Figure 1A**; **Supplementary Figures 1C, D**). To improve its pharmacokinetic properties, the LiTCo was fused to the N-terminus of an albumin variant with enhanced affinity for human FcRn (Albu-LiTCo) (22, 50). Both antibodies were efficiently expressed in biologically active form by transfected human embryonic kidney HEK293 cells. Western blot analysis under reducing conditions of cell culture supernatants (SNs) showed bands with migration patterns consistent with their estimated molecular weights (**Supplementary Figures 2A, C**). To assess target specificity, ELISA binding assays were performed. SN of LiTE-lipofected HEK293 cells specifically bound to mouse and human EGFR (**Supplementary Figure 2B**), while Albu-LiTCo-lipofected SN bound to mouse and human PD-L1, as well as mouse 4-1BB (**Supplementary Figures 2D, E**). One-step chromatography yielded ∼90% pure LiTE and Albu-LiTCo with post-purification yields of approximately 0.5 mg/L HEK293 SN. SEC analysis revealed major monomeric peaks of 47 kDa (LiTE) and 110 kDa (Albu-LiTCo), consistent with their theoretical molecular weights (**Supplementary Figures 3B, D**). ELISA showed dose-dependent binding of purified LiTE to EGFR (**Supplementary Figure 4A**) and purified Albu-LiTCo to PD-L1 and 4-1BB (**Supplementary Figures 4B, C**). Their ability to specifically recognize the antigens in a cellular context was analyzed by flow cytometry. LiTE bound to mouse CD3 on the T cell line 2B4 (**Supplementary Figure 5A**) and to human EGFR-expressing mouse CT26 cells (CT26^EGFR^) (**Supplementary Figure 5C**), but not to wild-type CT26 cells or human Jurkat T cells. As for Albu-LiTCo, it bound specifically to genetically engineered HEK293 cells to express mouse 4-1BB on their cell surface (HEK293^4-1BB^) (**Supplementary Figure 5B**) and to CHO cells expressing human PD-L1 (CHO^PD-L1^) (**Supplementary Figure 5D**). No binding to wild-type HEK293 or CHO cells was detected.

### LiTE induces EGFR-dependent T cell activation and cell killing

The functionality of purified LiTE was further assessed by analyzing T cell activation in cocultures of mouse splenocytes with CT26 or CT26^EGFR^ cells at an effector-to-target (E:T) ratios of 5:1. CD69 was expressed after 24 hours of coculture with CT26^EGFR^ cells, but not with wild-type CT26 cells, as determined by flow cytometry (**Figure 1B**). Purified LiTE directed T cells to lyse luciferase-expressing CT26^EGFR^ tumor cells in a dose-dependent manner when *in vitro* cocultured with murine splenocytes at a E:T ratio of 5:1 [median effective concentration (EC_50_), 15.4 nM] (**Figure 1C**), indicating that EGFR cross-linking is required for LiTE-induced cytotoxicity. No appreciable lysis of EGFR-negative CT26 tumor cells was detected at any concentration. Cytokine release (IFNγ and IL-2) was only observed when splenocytes were cocultured with CT26^EGFR^ cells (**Supplementary Figure 6**). Together, purified LiTE was highly monomeric and mediated robust T cell activation that was conditioned on both EGFR and mouse CD3 binding.

### Albu-LiTCo effectively blocks the PD-1/PD-L1 pathway

The blockade of the PD-L1/PD-1 pathway by Albu-LiTCo was evaluated by competition ELISA and compared to the bivalent anti-PD-L1 mAb atezolizumab. Albu-LiTCo effectively inhibited the PD-1/PD-L1 interaction with a half maximal inhibitory concentration (IC_50_) of 52.68 nM versus 10.20 nM for atezolizumab (**Figure 1D**). In cell-based assays using an NFAT-luciferase reporter Jurkat cell line expressing human PD-1, Albu-LiTCo efficiently blocked the PD-1/PD-L1 interaction, resulting in dose-dependent NFAT activation and light emission with an IC_50_ of 1.627 nM, slightly higher than that observed with atezolizumab (0.586 nM) (**Figure 1E**).

### Albu-LiTCo PD-L1-conditional 4-1BB agonist activity enhances EGFR-specific T cell activation mediated by LiTE

We studied the 4-1BB agonistic activity of Albu-LiTCo in co-cultures of isolated mouse CD8a^+^ T cells preactivated with LiTE at 0.175 μg/ml (3.34 nM) in the presence of wild-type CHO cells and CHO cells expressing human PD-L1 and human EGFR (CHO^PD-L1/EGFR^) (**Figure 1F**). Albu-LiTCo significantly increased IFNγ secretion of LiTE-activated T cells in co-culture with CHO^PD-L1/EGFR^ cells (P=0.0078) (**Figure 1F**), but not in co-culture with wild-type CHO cells. LiTE alone also increased IFNγ secretion following EGFR-specific crosslinking but at lower levels (p=0.0194) (**Figure 1F**). Addition of anti-4-1BB or anti-PD-L1 IgG antibodies also increased IFNγ levels when T CD8a^+^ cells were co-cultured with CHO^PD-L1/EGFR^ cells in the presence of LiTE (P=0.0020 and P=0.0139, respectively), but the effect was milder than that observed with Albu-LiTCo. IFNγ secretion was negligible in the absence of LiTE-mediated EGFR-specific crosslinking. Activation of CD8a^+^ T cells with soluble anti-mouse CD3 mAb (0.5 µg/ml) in the presence of CHO^PD-L1^ cells (**Supplementary Figure 7A**) or plastic-immobilized PD-L1 (**Supplementary Figure 7B**) also promoted significantly increased IFNγ levels (P = 0.0026 and P = 0.0067, respectively).

### mRNA-encoded LiTE and Albu-LiTCo bispecific antibodies are functionally active

**Figure 2:**
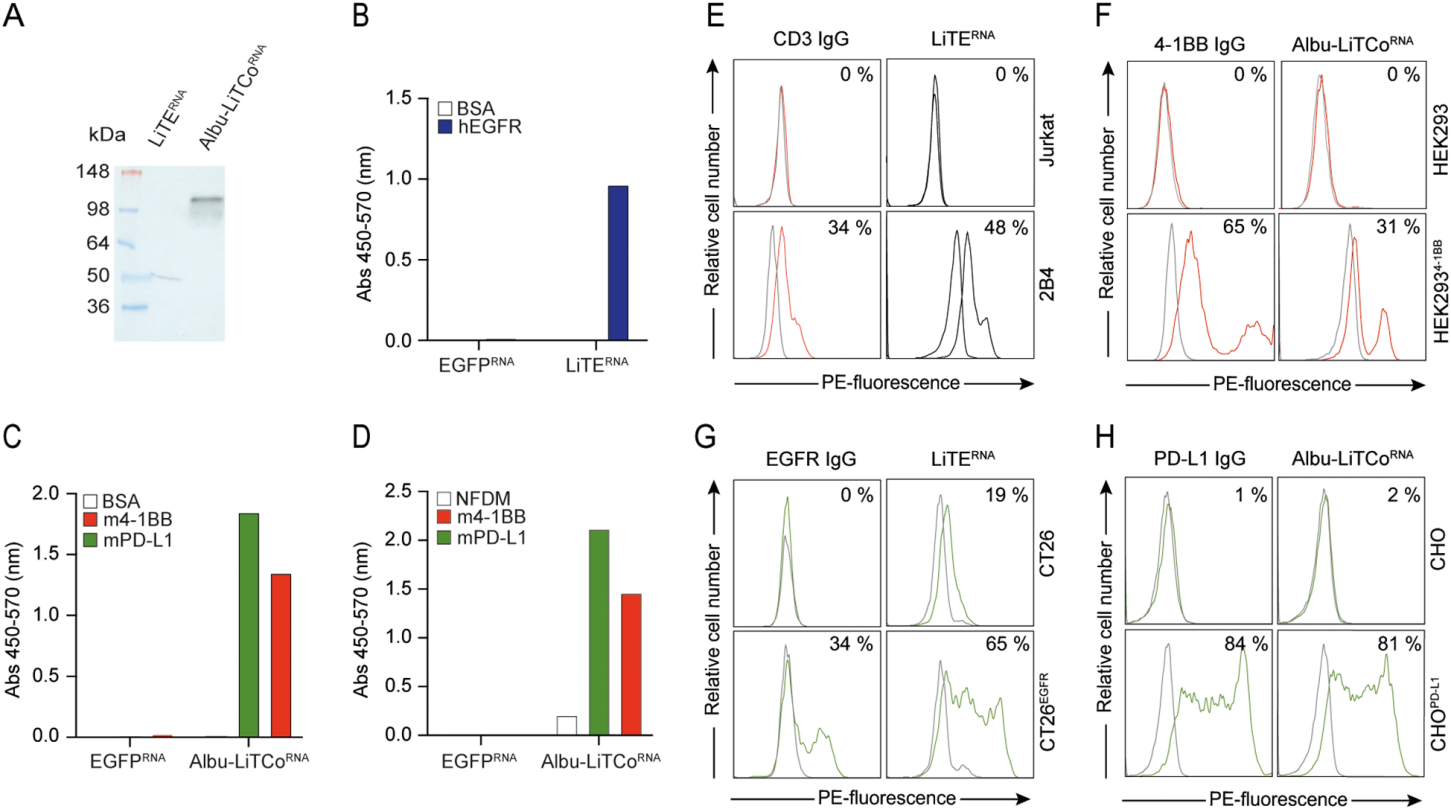
Functional characterization of LiTE^RNA^ and Albu-LiTCo^RNA^. Western blot detection using a HRP-conjugated anti-V_HH_ mAb in the conditioned media of LiTE^RNA^ and Albu-LiTCo^RNA^ transfected HEK293 cells. Molecular mass (kDa) is indicated **(A)**. Specific binding of LiTE^RNA^ **(B)** and Albu-LiTCo^RNA^ **(C, D)** to plastic immobilized specific antigens (hEGFR and m4-1BB, mPD-L1, respectively) demonstrated by ELISA using an HRP-conjugated anti-V_HH_ mAb cocktail **(B, C)** or HRP-conjugated anti-human serum albumin (HRP-HSA) mAb **(D)**. Data are represented as a mean ± SD (n = 3). BSA, bovine serum albumin; NFDM, non-fat dry milk. Binding of LiTE^RNA^ and Albu-LiTCo^RNA^ to cell surface expressed antigens mCD3 **(E)**, m4-1BB **(F)**, hEGFR **(G)** and hPDL-1 **(H)** by flow cytometry. The y-axis shows the relative cell number, and the x-axis represents the R-phycoerythrin (PE)-fluorescence, expressed on a linear scale. The anti-CD3 IgG, anti-4-1BB IgG, anti-EGFR IgG and anti-PD-L1 IgG were used as controls. One representative experiment out of two independent experiments is shown. The number indicates the percentage of positive cells (%).

**Figure 3:**
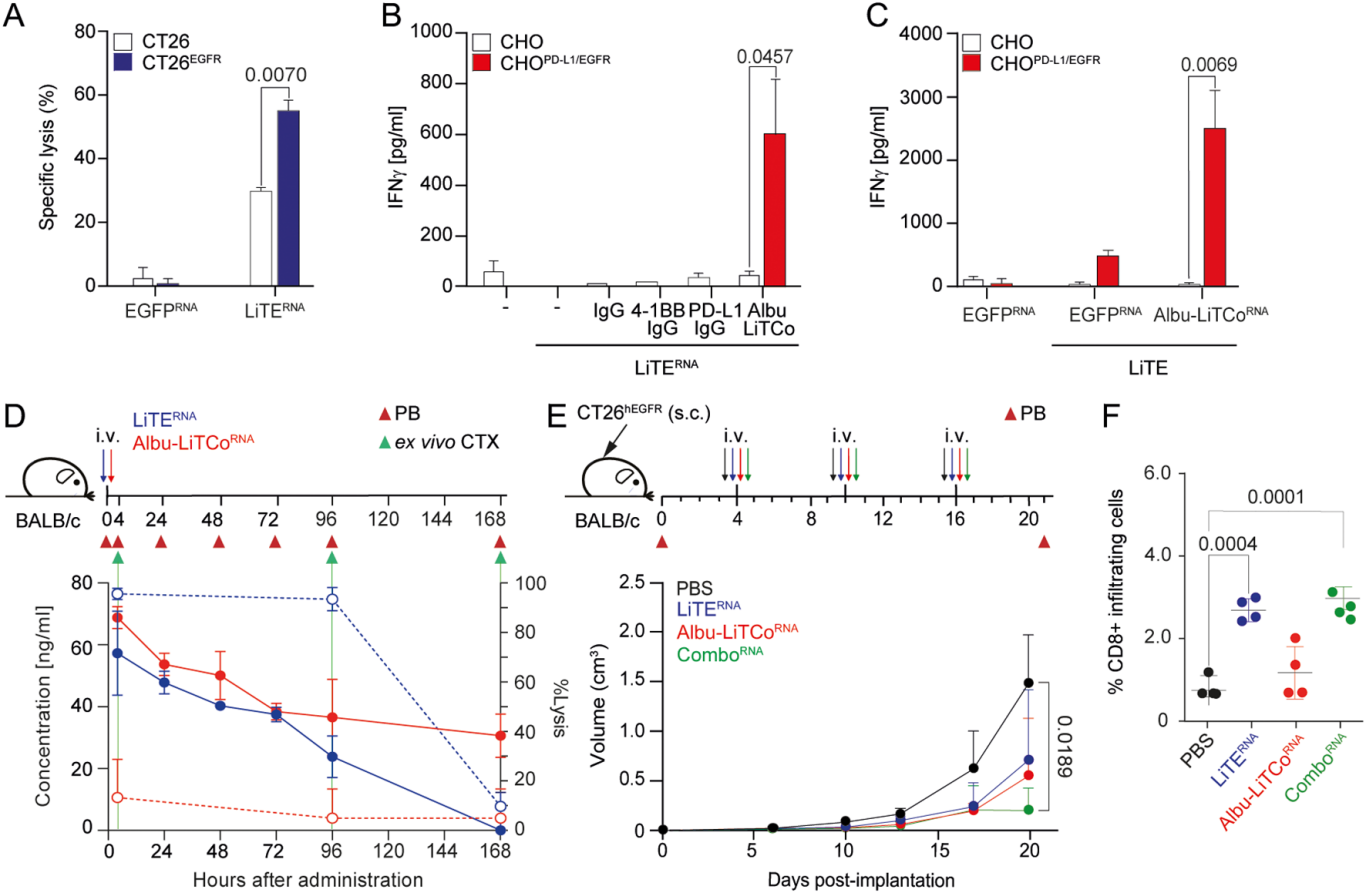
Effects of LiTE^RNA^ and Albu-LiTCo^RNA^ on tumor cell cytotoxicity, agonistic costimulatory activity and tumor growth inhibition. EGFR-dependent cytotoxicity by LiTE^RNA^ **(A)**. Luciferase-expressing CT26 and CT26^EGFR^ cells were co-cultured with mouse splenocytes at an E:T ratio of 5:1. Specific tumor cell lysis was measured by bioluminescence. The percentage of specific lysis was calculated relative to the same number of tumor cells cultured with splenocytes. Data are expressed as mean ± SD (n = 3). Significance was calculated by unpaired Student’s *t* test. CHO or CHO^PD-L1/EGFR^ cells were plated with mouse CD8a^+^ T cells activated with LiTE^RNA^ in the presence of Albu-LiTCo **(B)** or with LiTE in the presence of Albu-LiTCo^RNA^ **(C)**. IFNγ secretion was determined after 72 hours. Data are expressed as mean ± SD (n = 3). Significance was calculated by unpaired Student’s *t* test. Anti-4-1BB IgG and anti-PD-L1 IgG **(B)** or EGFP^RNA^ **(C)** were used as controls. Pharmacokinetic profile of LiTE^RNA^ (solid blue line) and Albu-LiTCo^RNA^ (solid red line) expressed as ng/mL (left Y-axis) after a single intravenous (i.v.) administration of mRNA-LNP in BALB/c mice **(D)**. *Ex vivo* specific tumor lysis of CT26^EGFR^ cells mediated by LiTE-containing mouse serum (dashed blue line) and Albu-LiTCo-containing mouse serum (dashed red line) expressed as % of Lysis (right Y-axis) **(D)**. Target cells modified for the expression of luciferase were co-cultured with splenocytes at the effector/target (E/T) ratio of 5:1 in the presence of mouse serum obtained at 4, 96 and 168 hours post-mRNA-LNP administration. After 48 hours, the percentage of specific tumor lysis was measured by bioluminescence (right Y-axis). Data are presented as mean ± SD (n = 3). BALB/c mice were subcutaneously (s.c.) inoculated with CT26^EGFR^ cells, randomized into n=5/group with similar mean tumor sizes and SDs and treated with 3 i.v. injections of LiTE^RNA^, Albu-LiTCo^RNA^ (10 µg/mouse) as monotherapy or combined (Combo^RNA^) **(E)**. Average tumor volume growth of mice in each group is shown. Data are presented as mean ± SD. Significance was determined by one-way ANOVA with Tukey’s correction test for multiple comparisons. Quantitative analysis of intratumoral CD8^+^ T cells in mouse tumor tissue (n = 4/group) **(F)**. Data were calculated as percentage of CD8^+^ versus total cell number and presented as mean ± SD. Significance was determined by one-way ANOVA with Tukey’s test correction for multiple comparisons.

The mRNA-encoding bispecific antibodies contain a 5’ type I cap (5’ Cap: Cap1) and N1-methylpseudouridine modified nucleosides (N1-me-Ψ). The mRNA template includes a 5’ UTR, Kozak sequence, 3’ UTR and poly(A) tail, as well as the LiTE or Albu-LiTCo coding sequences. We first tested whether HEK293 cells transfected with mRNA expressed functional versions of both bispecific antibodies. Bands of the expected molecular weight were detected by Western blot in SN of mRNA-transfected HEK293 cells (**Figure 2A**). In addition, specific binding to hEGFR by LiTE^RNA^ (**Figure 2B**) and to mPD-L1 and m4-1BB by Albu-LiTCo^RNA^ (**Figures 2C, D**) was demonstrated by ELISA. Correspondingly, flow cytometry analysis showed binding of LiTE^RNA^ to mouse CD3 on 2B4 T cells and to human EGFR on CT26^EGFR^ cells (**Figures 2E, G**), as well as binding of Albu-LiTCo^RNA^ to stably transfected cells expressing m4-1BB or hPD-L1 (**Figures 2F, H**). No binding was observed on untransfected cells. LiTE^RNA^ (3.3 µg/ml) elicited a strong cytotoxic response in cocultures with murine splenocytes and CT26^EGFR^ cells at an E:T ratio of 5:1 (P=0.019) (**Figure 3A**), with an efficacy comparable to that of the corresponding recombinant purified LiTE at the highest concentration used (**Figure 1C**). In addition, CD8a^+^ T cells preactivated with LiTE^RNA^ (3.3 µg/ml) (**Figure 3B**) or purified LiTE (3.34 nM) (**Figure 3C**) in the presence of purified Albu-LiTCo (6.67 nM) or Albu-LiTCo^RNA^ (3.9 µg/ml), respectively, showed a significant increase in IFNγ levels when cocultured with CHO^PDL1/EGFR^ cells but not with CHO cells (**Figures 3B, C**).

### Pharmacokinetics of mRNA-encoded LiTE and Albu-LiTCo

LiTE^RNA^ and Albu-LiTCo^RNA^ were formulated with a polymer/lipid-based nanoparticle (LNP) to ensure efficient translation in the liver following intravenous (i.v.) administration (40). Following a single dose of 10 µg of mRNA-LNP, translated proteins peaked within 4 hours (approximately 100 ng/ml). Protein levels remained at 40 ng/ml for 72 hours in the case of LiTE^RNA^ and 30 ng/ml for up to 168 hours in the case of Albu-LiTCo^RNA^ (**Figure 3D**). As previously reported, the engineered human albumin^HB^ fused to the LiTCo molecule significantly enhanced its PK properties (22). In addition, *in vivo* production of the bispecific antibodies promoted significantly longer circulating half-lives (LiTE; terminal t_1/2_, 18.9 ± 0.018 hours; Albu-LiTCo; terminal t_1/2_, 49.62 ± 0.050 hours) compared to purified protein (**Supplementary Figure 8**) (22). Purified LiTE exhibited rapid clearance from the circulation, with a terminal half-life of just 0.59 ± 0.001 hours, whereas the Albu-LiTCo demonstrated a significantly extended circulatory half-life of 22.15 ± 0.017 hours (**Supplementary Figure 8**). To confirm the cytotoxicity potential of *in vivo* produced LiTE, serum from mice that had received 10 µg of mRNA-LNP LiTE^RNA^ or Albu-LiTCo^RNA^ was added to cocultures of mouse splenocytes with either EGFR-positive (CT26^EGFR^) or EGFR-negative (CT26) cells at an E:T ratio of 5:1. LiTE-containing mouse serum collected at 4 hours and 4 days after LiTE^RNA^-LNP i.v. administration mediated EGFR-specific cytotoxicity of CT26^EGFR^ tumor cells *ex vivo*, indicating full functionality of the antibody produced *in vivo* (dashed blue line) (**Figure 3D**). A correlation between serum LiTE levels and specific cytotoxic activity was observed. No significant lysis of EGFR-expressing cells was detected in cocultures with mouse serum collected on day 7 (**Figure 3D**). No cytotoxicity was observed in cocultures with Albu-LiTCo mouse serum at any time point (dashed red line) (**Figure 3D**). Lysis of EGFR-negative CT26 tumor cells was negligible in the presence of serum containing either LiTE- or Albu-LiTCo (**Supplementary Figure 9**).

### Combined treatment with mRNA-encoded LiTE and Albu-LiTCo slows tumor growth without signs of toxicity

After demonstrating that pharmacologically active levels of both bispecific antibodies could be achieved by i.v. mRNA-LNP administration, we investigated their antitumor activity in an immunocompetent mouse model subcutaneously xenografted with CT26^EGFR^ colorectal tumors (10, 22), which constitutively expresses low levels of PD-L1 that increase in response to soluble IFNγ (**Supplementary Figure 10**). Mice were treated once a week with i.v. injection of 10 μg LiTE mRNA-LNP (LiTE^RNA^), Albu-LiTCo mRNA-LNP (Albu-LiTCo^RNA^), the combination (Combo^RNA^) or PBS for 3 weeks. Both LiTE^RNA^ and Albu-LiTCo^RNA^, when administered as monotherapy, resulted in a delayed tumor growth that was not statistically significant. However, the therapeutic effect of Combo^RNA^ was superior to both monotherapies, leading to a significant reduction in tumor growth (P = 0.0189) (**Figure 3E**). In addition, the Combo^RNA^ achieved at least one complete regression out of five mice bearing CT26^EGFR^ tumors (**Supplementary Figure 11D**). Neither treatment resulted in the development of splenomegaly or hepatomegaly (**Supplementary Figures 12A, B**), and IFNγ levels evaluated 96 hours after the second injection were comparable to those found in PBS-treated mice (**Supplementary Figure 12C**). To quantify lymphocyte infiltration immunohistochemical (IHC) staining of mouse CD8 was performed on tumor xenografts. CD8^+^ T cells increased significantly in tumors treated with LiTE^RNA^ and Combo^RNA^ (P = 0.004 and P = 0.001, respectively) (**Figure 3F**; **Supplementary Figure 12D**).

## Discussion

In this study, we combine for the first time two mRNA-encoded bispecific antibodies with complementary mechanisms of action and demonstrate their potential to induce effective antitumor responses with a favorable toxicity profile. We designed and characterized two V_HH_-scFv tandem bispecific antibodies. The anti-EGFR x anti-CD3 LiTE specifically activates and redirects polyclonal mouse T cells to kill EGFR-positive cancer cells, while the Albu-LiTCo combines PD-L1/PD-1 axis blockade with PD-L1-dependent 4-1BB costimulation and enhances EGFR-specific LiTE T cell-mediated activation *in vitro*.

A disadvantage of Fc-free, fragment-based bispecific antibodies administered as purified proteins is their short half-life and the need for frequent dosing or continuous infusion to achieve sustained therapeutic concentrations, which severely limit their clinical potential (51, 52). In this context, mRNA-based delivery of therapeutic antibodies may be a promising approach (53–55) as it allows *in vivo* expression, avoiding the complex manufacturing process of proteins and potentially increasing serum half-life, and achieves remarkable therapeutic effects (40, 56–58). Here, we implemented a polymer/lipid-based formulation for the systemic delivery of mRNA-encoded bispecific antibodies (40). *In vitro*-translated LiTE^RNA^ and Albu-LiTCo^RNA^ were shown to have comparable biological functionalities to purified proteins, and after i.v. administration of mRNA-LNP, pharmacokinetic analysis showed that this approach resulted in sustained *in vivo* production of both bispecific antibodies. Given the favorable toxicity profile of Albu-LiTCo, we employed a HLE strategy to prolong its circulation time, thereby increasing the probability of reaching the tumor and loco-regional lymph nodes, to block the PD-1/PD-L1 axis and provide PD-L1-specific 4-1BB costimulation to induce the proliferation and survival of antigen primed tumor-reactive T cells. Albu-LiTCo was fused to albumin^HB^, a human variant sequence with high avidity for FcRn (22, 37), with a half-life of 49.62 hours after mRNA-LNP administration. For inducing polyclonal T cell engagement, we designed an albumin-free LiTE molecule with a half-life of 0.59 hours when administered as a purified protein and of 18.9 hours after mRNA-LNP delivery, ensuring a shorter systemic exposure cycle compared to Albu-LiTCo and minimizing on-target off-tumor toxicity. In fact, although the anti-EGFR V_HH_ recognizes mouse EGFR, no significant adverse effects were observed during treatment for 3 weeks with LiTE as mRNA monotherapy or combo with Albu-LiTCo. The use of Albu-LiTCo^RNA^ as monotherapy results in limited antitumor responses *in vivo*. PD-L1 has been shown to be upregulated on tumor, stromal and immune cells upon IFNγ release (59–61). TCE-mediated T cell activation could lead to an increase in PD-1 expression, thereby reducing treatment efficacy (35, 61, 62). Co-administration of TCE with ICIs has shown improved tumor control and synergistic effects in numerous preclinical studies (17, 62–65). Consistent with this, our study demonstrated that Combo^RNA^ treatment induced a significantly enhanced antitumor response even at low serum levels of both bispecific antibodies (in the ng/ml range). Furthermore, our results showed that LiTE^RNA^ was able to increase intratumoral CD8^+^ T cell infiltration both as monotherapy and in combination, but the co-administration of Albu-LiTCo^RNA^ effectively improved T cell functionality. In addition, the Combo^RNA^ demonstrated effective tumor suppression without associated toxic effects (30, 66, 67).

In conclusion, our results suggest that combined *in vivo* delivery of two mRNAs encoding an EGFR-specific TCE and a PD-L1-dependent 4-1BB agonist is a safe and feasible strategy to induce antitumor responses. The tumor model used was designed to evaluate the biological activity and potential efficacy of the mRNA-based strategy in a controlled setting, rather than to replicate the clinical context of advanced tumors. Future studies will focus on optimizing the dosing regimen and evaluating the long-term efficacy of the treatment.

## Data Availability

The original contributions presented in the study are included in the article/**Supplementary Material**. Further inquiries can be directed to the corresponding authors.
