# Supplementary material for: Effective cancer immunotherapy combining mRNA-encoded bispecific antibodies that induce polyclonal T cell engagement and PD-L1-dependent 4-1BB costimulation
Source: Front Immunol. 2025 Jan 6;15:1494206. doi: 10.3389/fimmu.2024.1494206 (PMC11743637; doi:10.3389/fimmu.2024.1494206)
Supplement: Supplementary file 1 [file DataSheet1.docx]

**Supplementary Material**

**Effective cancer immunotherapy combining mRNA-encoded bispecific antibodies that induce polyclonal T cell engagement and PD-L1-dependent 4-1BB costimulation**

Oana Hangiu^1,2,3^, Rocío Navarro^1^, Susana Frago^1^, Laura Rubio-Pérez^2,3,4^ , Antonio Tapia-Galisteo^2,3,4^, Laura Díez^2,3,4^, Marina Gómez-Rosel^1,2,3^, Noelia Silva-Pilipich^5,6^, Lucía Vanrell^7^, Cristian Smerdou^5,6^, Kenneth A. Howard^8^, Laura Sanz^9^, Luis Álvarez-Vallina^2,3,4^ and Marta Compte^1^


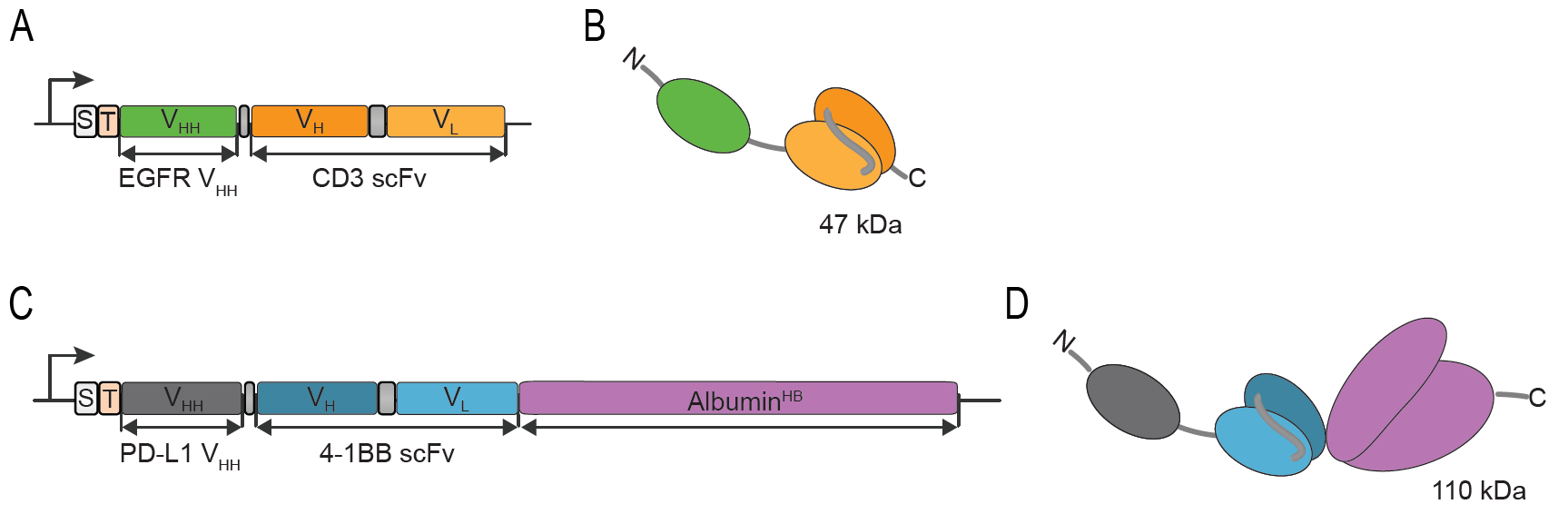


**Figure S1**. **Schematic diagrams showing the gene layout (A, C) and protein structure (B, D) of EGFR-targeted human light T cell engager (LiTE) (A, B) and PD-L1-targeted light T cell costimulatory (Albu-LiTCo) (C, D) antibodies**. The LiTE gene construct (**A**) contains an EGFR-specific V_HH_ domain (green) linked by a flexible linker (light grey boxes) to an anti-CD3 scFv (V_H_-linker-V_L_) (orange). In the Albu-LiTCo (**C**), a PD-L1-specific V_HH_ (dark grey) is linked to an anti-4-1BB scFv (V_H_-linker-V_L_) (blue) and fused to an engineered albumin variant (purple) with enhanced binding ability to human FcRn (Albumin^HB^) expressed as a single polypeptide. The oncostatin M signal peptide (S) (white box) is used to direct secretion of the recombinant antibody fusions, and the FLAG-strepII tag (T) (light orange box) is attached for immunodetection and affinity purification. Arrows indicate transcription direction.


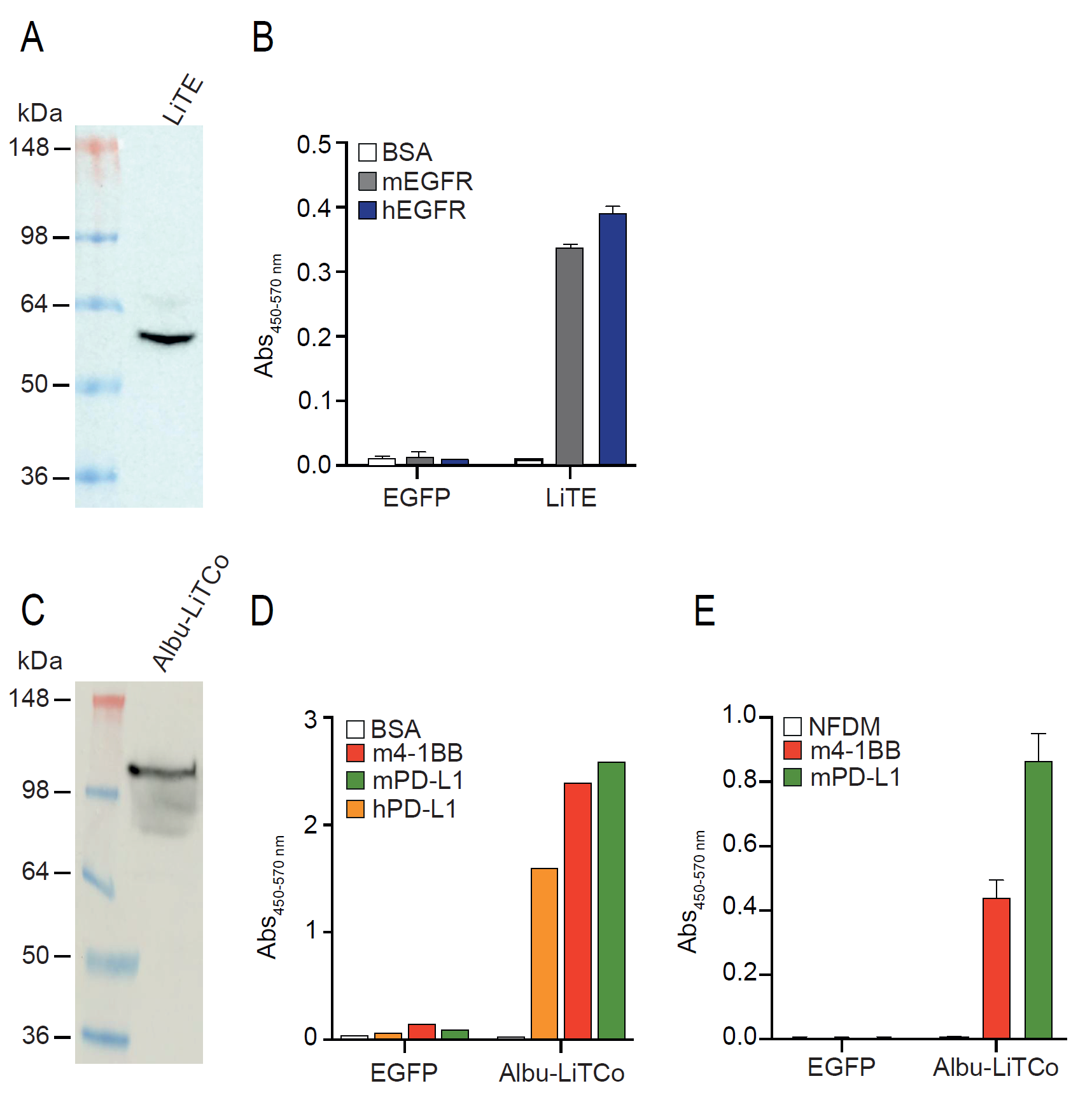


**Figure S2. Characterization of LiTE and Albu-LiTCo antibodies in supernatant from transfected HEK293 cells.** The presence of secreted LiTE (**A**) and Albu-LiTCo (**C**) in the cell culture supernatants was detected by Western blotting analysis developed with HRP-conjugated anti-V_HH_ mAb cocktail. Molecular mass marker is indicated (kDa). Functionality and specificity of secreted LiTE and Albu-LiTCo were demonstrated by ELISA against mouse (m) and human (h) EGFR (**B**), mouse (m) and human (h) PD-L1 and mouse (m) 4-1BB using an anti-FLAG mAb (**D).** Albu-LiTCo binding to m4-1BB and mPD-L1 was also assessed using an HRP-conjugated anti-human serum albumin (HSA-HRP) antibody **(E).** Data are expressed as a mean ± SD (*n* = 3). BSA, bovine serum albumin; NFDM, non-fat dry milk. The EGFP-conditioned media was used as a negative control.


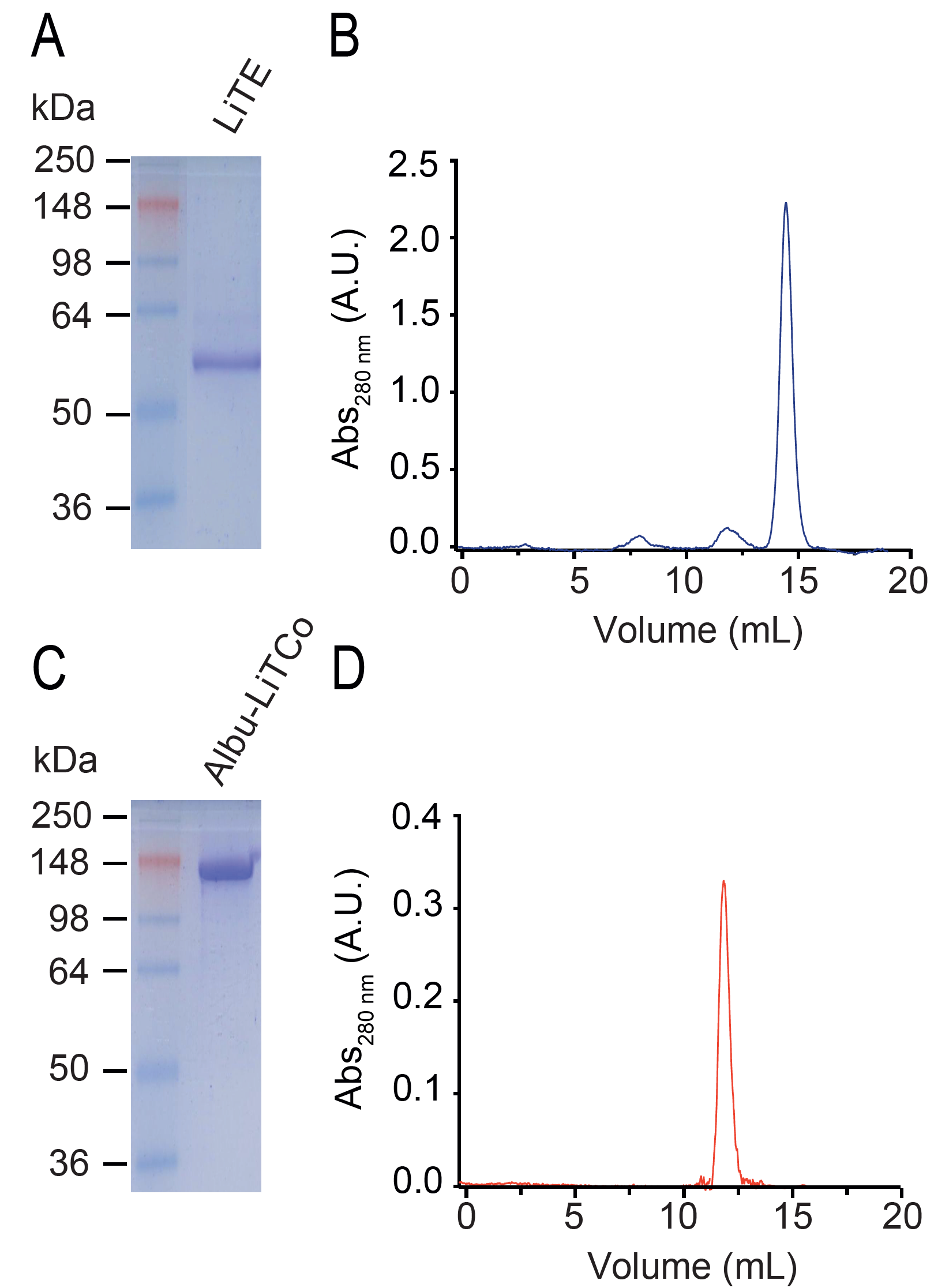


**Figure S3. Structural characterization of purified LiTE and Albu-LiTCo antibodies.** Reducing SDS-PAGE of purified LiTE (**A**) and Albu-LiTCo (**C**). Molecular mass marker is indicated (kDa). Size-exclusion analysis (SEC) of LiTE **(B)** and Albu-LiTCo **(D)** in PBS pH 7.4 at room temperature. Y-axis corresponds to the UV absorbance.


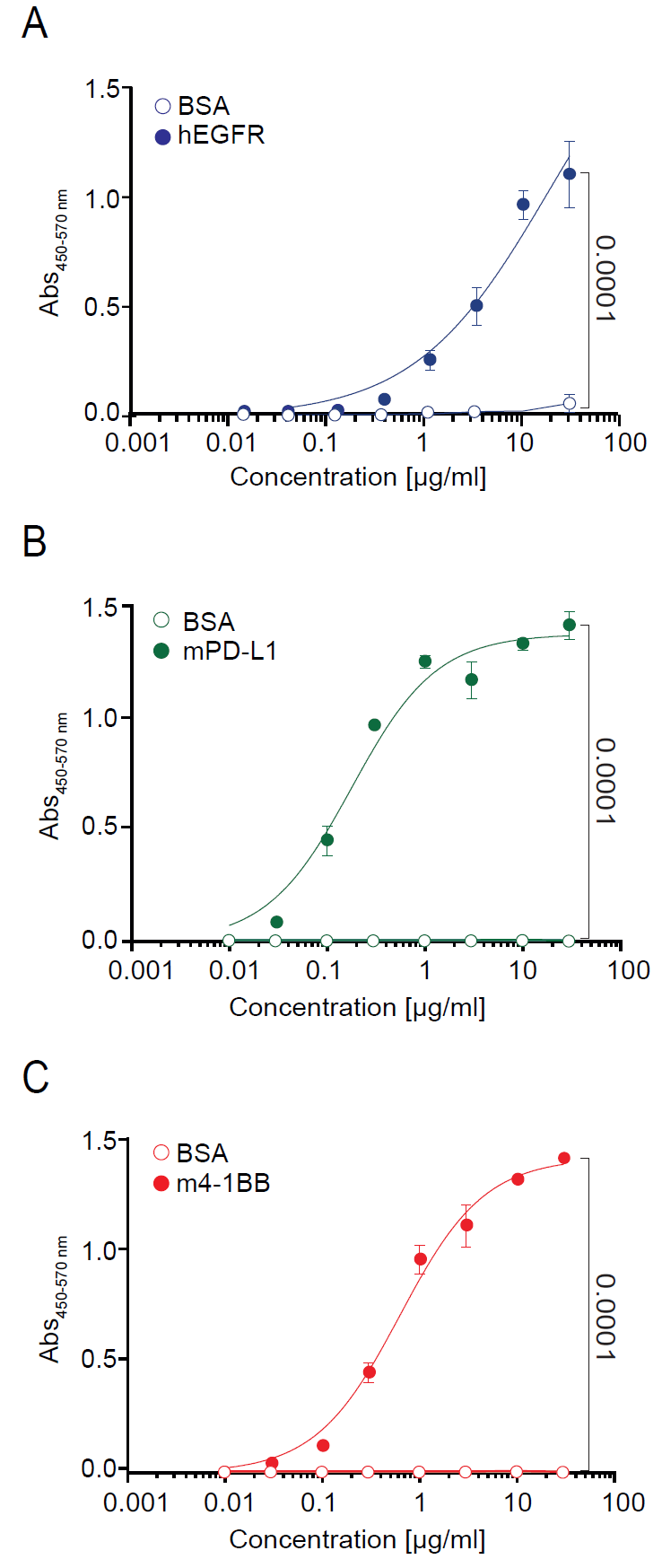


**Figure S4. Functional characterization of purified LiTE and Albu-LiTCo antibodies.** Saturation-binding curves with increasing concentrations of LiTE against plastic-immobilized hEGFR **(A),** and Albu-LiTCo against mPD-L1 **(B)** and m4-1BB **(C)**. Data are presented as mean ± SD (n = 3) of one representative experiment. Significance was calculated by an unpaired Student *t* test.


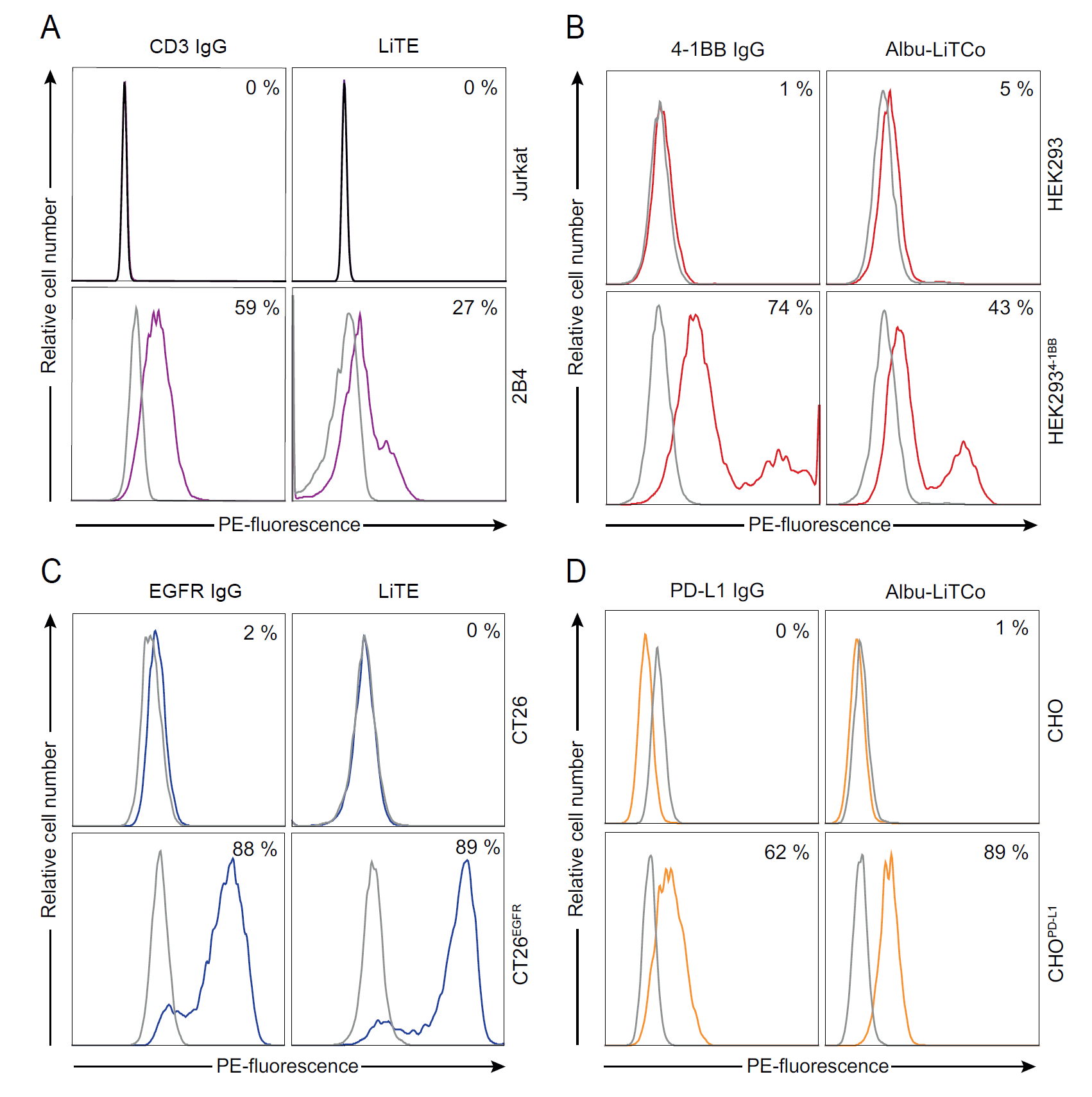


**Figure S5. Binding of purified LiTE and Albu-LiTCo antibodies to cell surface expressed antigens by flow cytometry.** The y-axis represents the relative cell number, and the x-axis represents the R-phycoerythrin (PE)-fluorescence, expressed on a linear scale. Anti-CD3 (2C11) IgG, anti-4-1BB (3H3) IgG, anti-EGFR (cetuximab) IgG, and anti-PD-L1 (atezolizumab) IgG were used as controls. One representative experiment of three independent experiments is shown. The number indicates the percentage of positive cells (%).


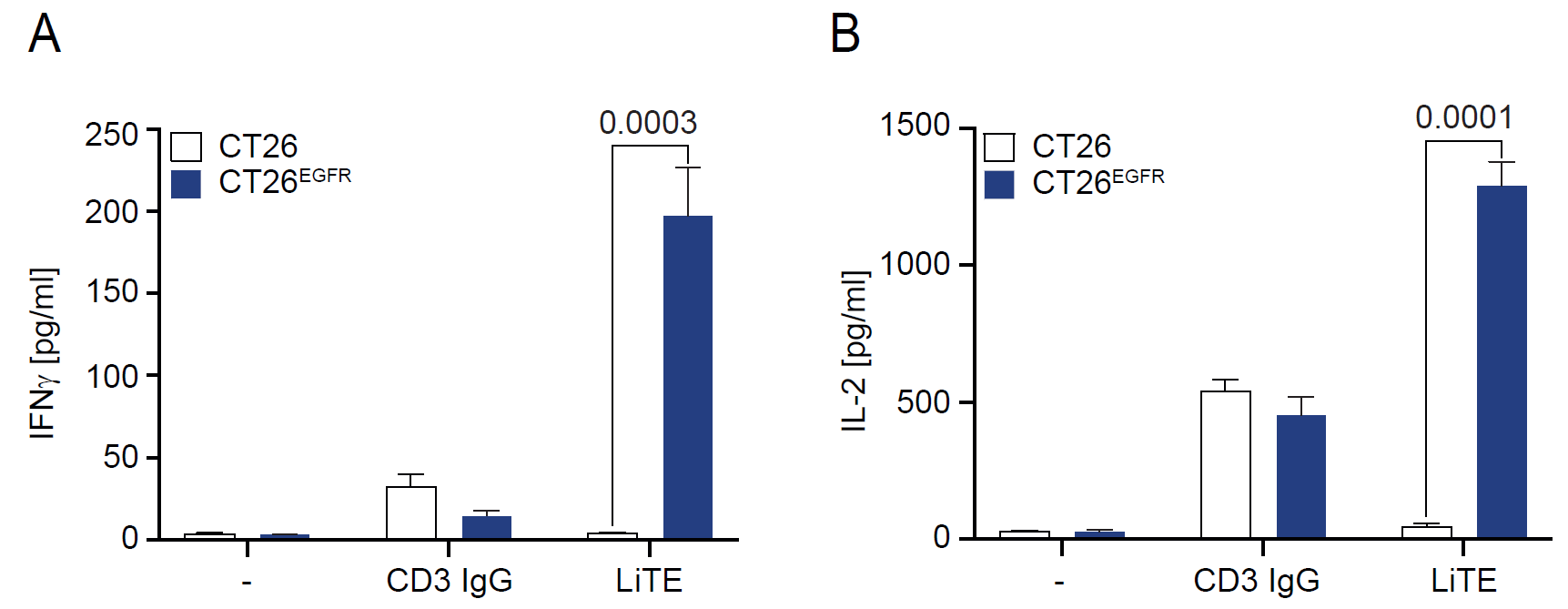


**Figure S6. Activation assay.** BALB/c mouse splenocytes were co-cultured with CT26 or CT26^EGFR^ cells in the presence of LiTE or anti-CD3 IgG. Levels of IFNγ (A) and IL-2 (B) were measured by ELISA after 24 hours. Data are expressed as mean ± SD (n = 3). Significance was determined by unpaired Student *t* test.


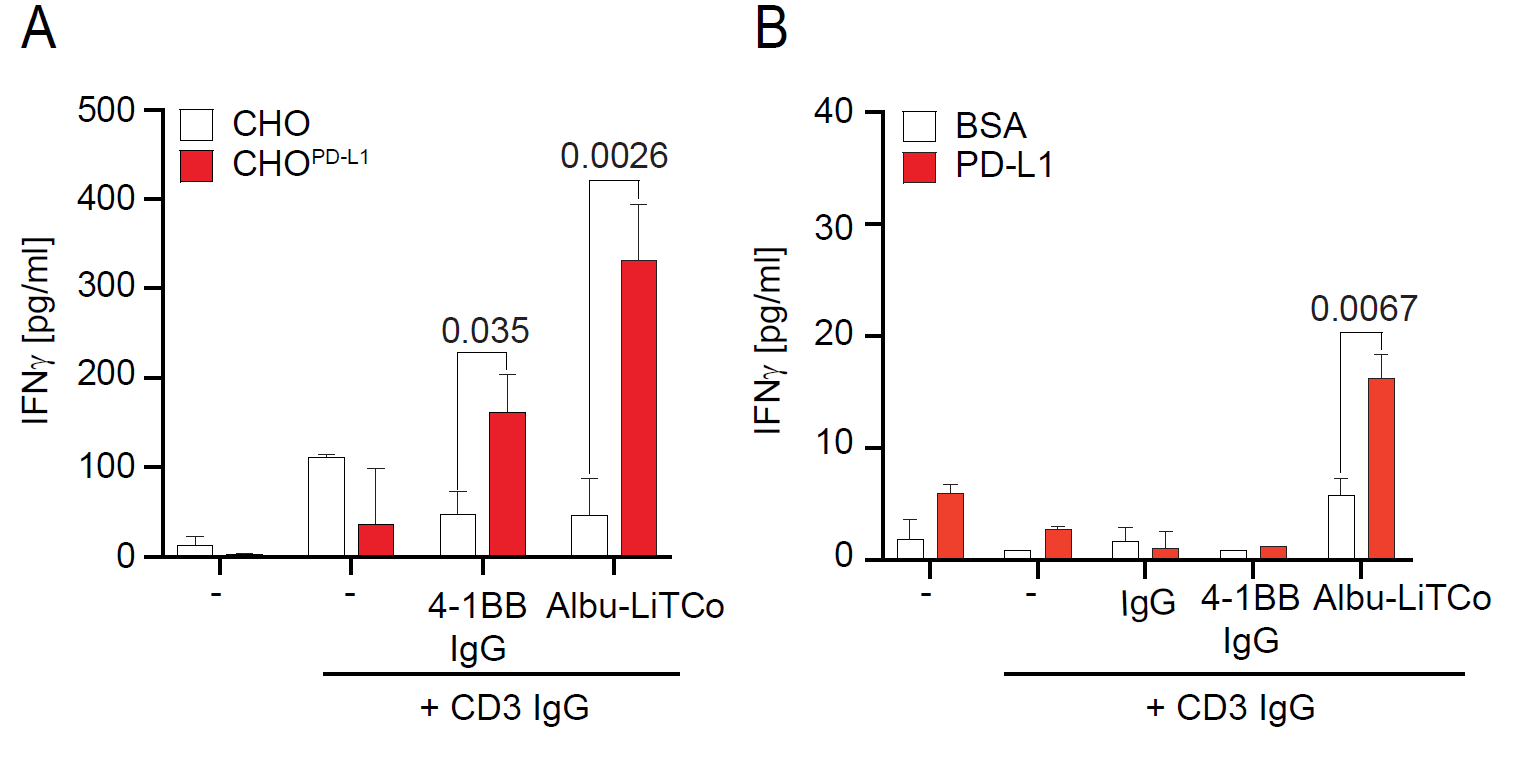


**Figure S7. Costimulatory activity.** CHO or CHO^PDL1^ cells were co-cultured with mouse isolated primary CD8a^+^ T cells activated with soluble anti-CD3 IgG (3.34 nM) in the presence of Albu-LiTCo or anti-4-1BB IgG at 6.67 nM equimolar dose **(A)**. Mouse CD8a^+^ T cells were plated with immobilized BSA or PD-L1 and activated with soluble anti-CD3 IgG (3.34 nM) in the presence of Albu-LiTCo, anti-4-1BB IgG or isotype IgG at 6.67 nM equimolar dose (**B**). IFNγ secretion was determined by ELISA after 72 hours. In both assays, negative controls (-) consisted of CD8^+^ T cells cultured either with or without PD-L1, and in the absence (- control in the left) or presence (- control in the right) of soluble anti-CD3 IgG. Data are expressed as mean ± SD (n = 3). Significance was calculated by an unpaired Student *t* test.


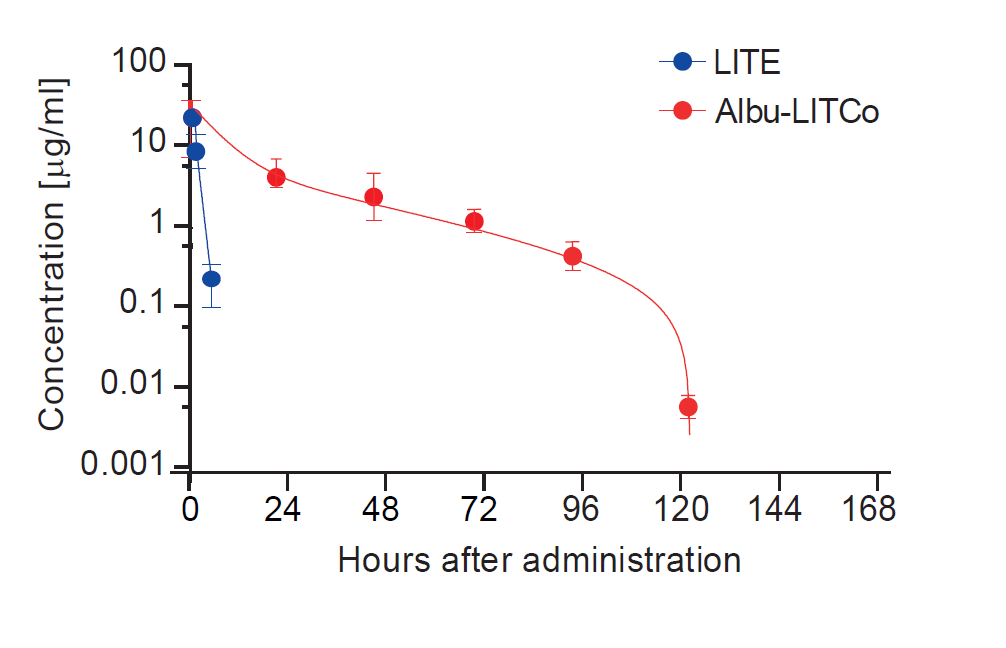


**Figure S8**. Pharmacokinetic study after a single intravenous (i.v.) dose of purified (1 mg/kg) LiTE (n = 4) and Albu-LiTCo (n = 4) in BALB/c mice. Data are shown as mean ± SD


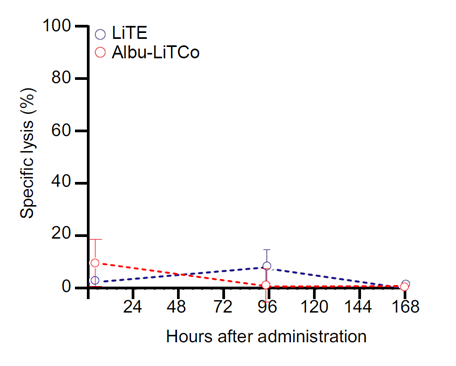


**Figure S9.** *Ex vivo* specific tumor lysis of EGFR-negative tumor cells. CT26 tumor cells co-cultured with splenocytes at an effector/target ratio of 5:1 in the presence LiTE- and Albu-LiTCo-containing mouse serum obtained at 4, 96 and 168 hours post-mRNA-LNP administration. After 48 hours, the percentage of specific tumor lysis was measured by bioluminescence. Data are presented as mean ± SD (n = 3).


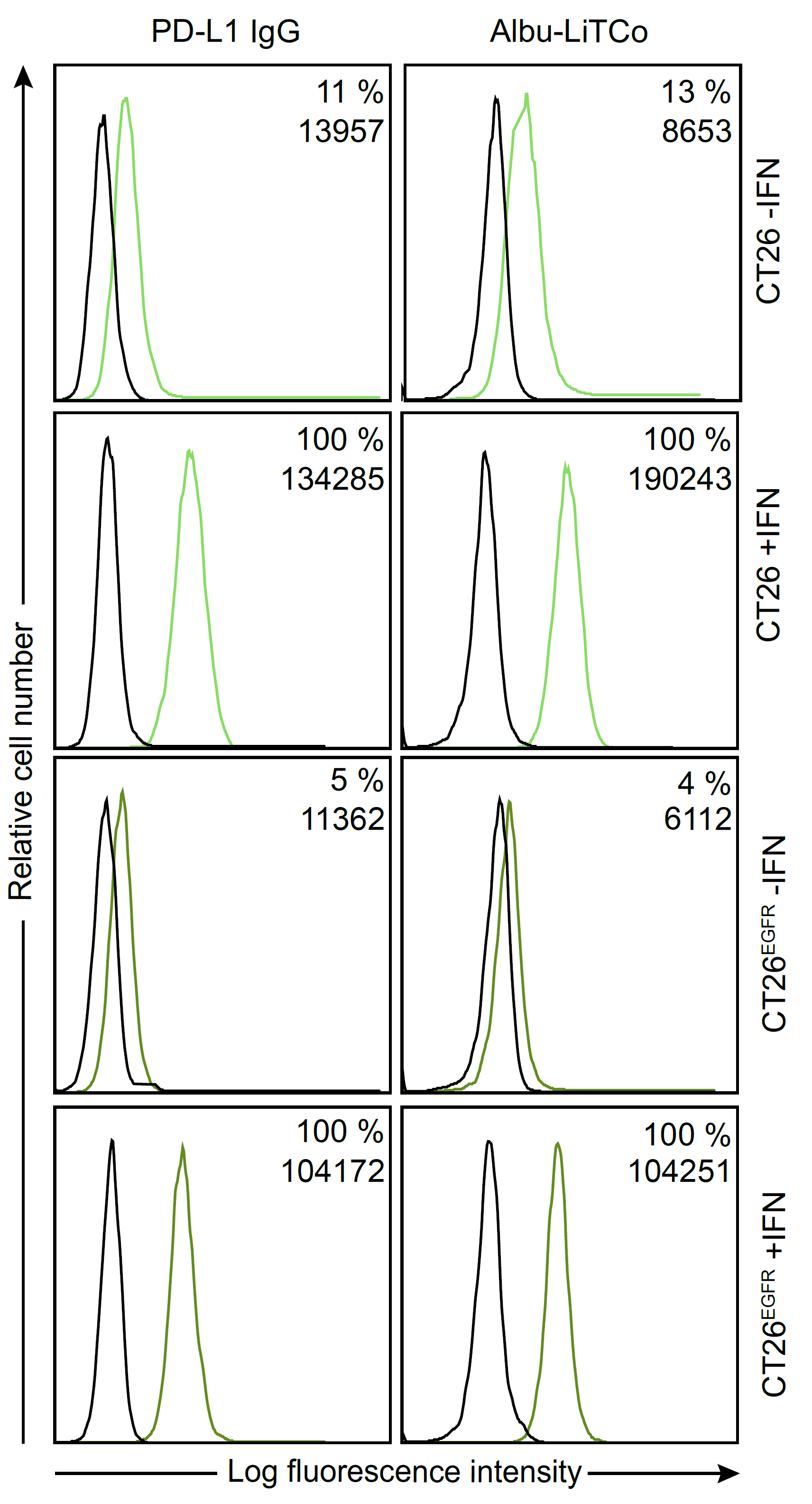


F**igure S10. Analysis of the expression of PD-L1** **on CT26 and CT26^EGFR^ cell surface.** CT26 and CT26^EGFR^ cells were incubated with or without soluble IFNγ (20 ng/ml) for 24 hours. Binding of PD-L1 IgG and Albu-LiTCo to PD-L1 was analyzed by flow cytometry. The y-axis shows the relative cell number, and the x-axis represents the intensity of fluorescence, expressed on a logarithmic scale. The numbers indicate the percentage of positive cells (%) and the mean fluorescence intensity (MFI).


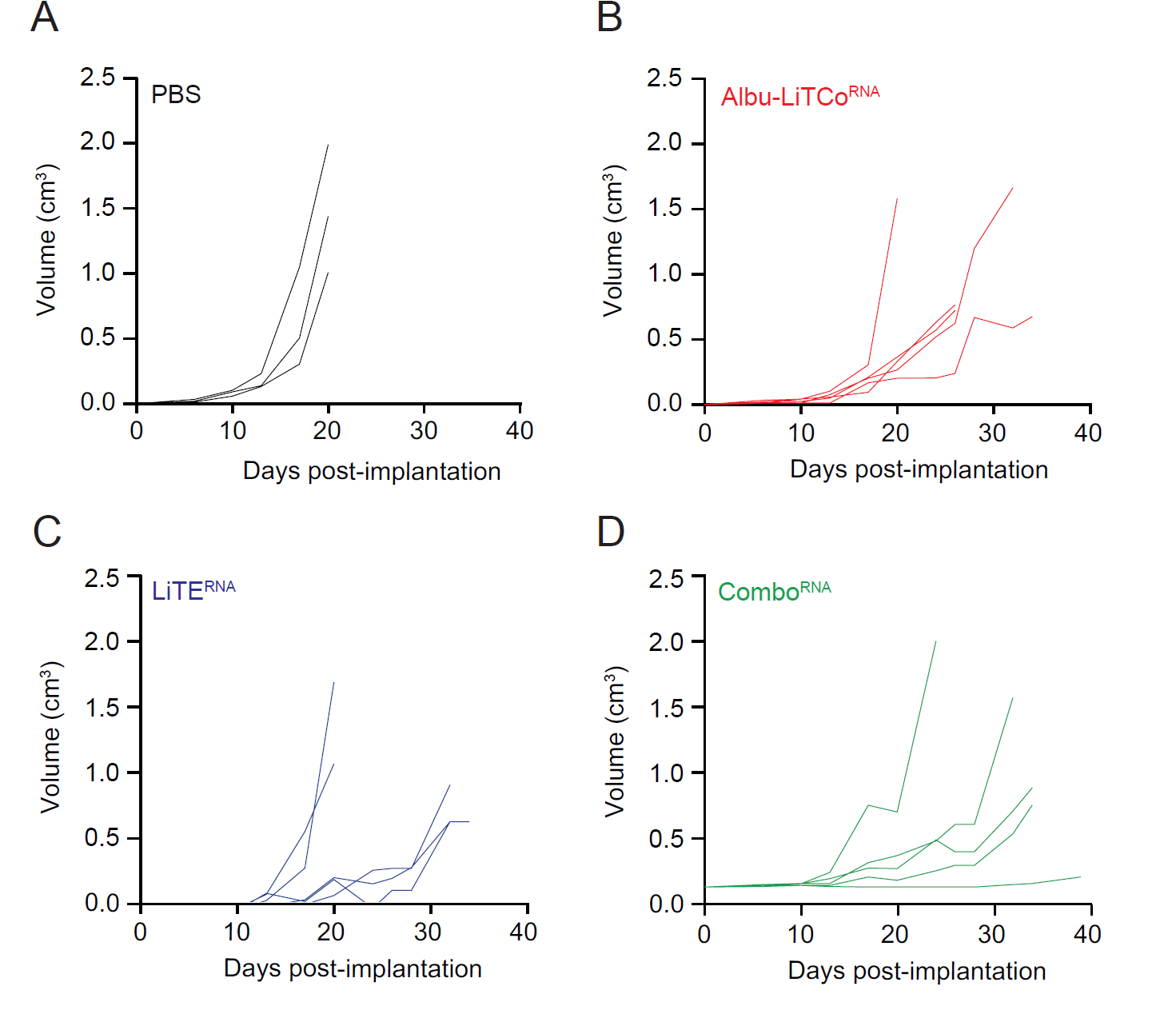


**Figure S11. Therapeutic assay.** BALB/c mice (n = 3-5/group) were subcutaneously inoculated with CT26^EGFR^ tumor cells and treated with 10 µg Albu-LiTCoRNA, LiTERNA or ComboRNA once a week for a total of three weeks. A control group received PBS. Mice were monitored every two days for tumor growth. Tumor volume growth curves are shown for individual mice in each treatment group.


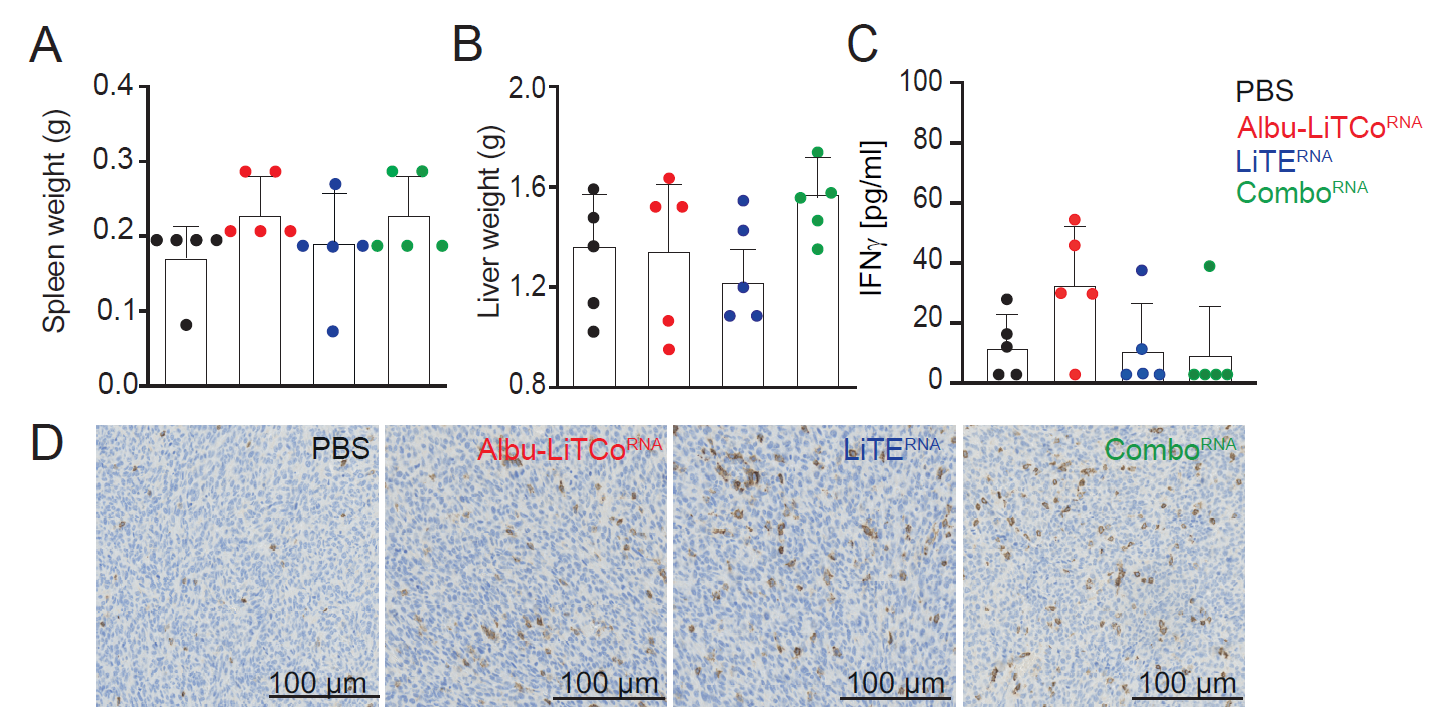


**Figure S12**. **Toxicity and immunohistochemistry analysis.** Spleen (**A**) and liver (**B**) were harvested, observed and weighted at the end of the experiment shown in Figure 2F. Significance was calculated by unpaired Student's t-test. Mouse IFNγ serum levels were analyzed (mean ± SD, *n* = 4-5) (**C**). Significance was calculated by unpaired Student's t-test. Representative images of immunohistochemical (IHC) staining of infiltrating CD8^+^ TILs in immunocompetent BALB/c mice bearing EGFR^+^ CT26 tumor cells treated with PBS, Albu-LiTCo^RNA^, LiTE^RNA^, and Combo^RNA^ (D).

**Table S1. Oligonucleotides used in this study**

| **Name** | **Sequence (5´-3´)** |
| --- | --- |
| FwCMV | CGCAAATGGGCGGTAGGCGTG |
| RvBGH | TAGAAGGCACAGTCGAGG |

Oligonucleotides were synthesized by Thermo Scientific.
